# Designing Weights for Quartet-Based Methods When Data are Heterogeneous Across Lineages

**DOI:** 10.1007/s11538-023-01167-y

**Published:** 2023-06-13

**Authors:** Marta Casanellas, Jesús Fernández-Sánchez, Marina Garrote-López, Marc Sabaté-Vidales

**Affiliations:** 1 Institut de Matematiques de la UPC-BarcelonaTech (IMTech), Universitat Politècnica de Catalunya and Centre de Recerca Matemàtica, Av. Diagonal 647, 08028 Barcelona, Spain; 2 University of British Columbia, Vancouver, Canada; 3 The University of Edinburgh, Edinburgh, UK

**Keywords:** Algebraic methods for topology reconstruction, Quartet-based methods, General Markov model, Paralinear method, Heterogeneity across lineages

## Abstract

Homogeneity across lineages is a general assumption in phylogenetics according to which nucleotide substitution rates are common to all lineages. Many phylogenetic methods relax this hypothesis but keep a simple enough model to make the process of sequence evolution more tractable. On the other hand, dealing successfully with the general case (heterogeneity of rates across lineages) is one of the key features of phylogenetic reconstruction methods based on algebraic tools. The goal of this paper is twofold. First, we present a new weighting system for quartets (ASAQ) based on algebraic and semi-algebraic tools, thus especially indicated to deal with data evolving under heterogeneous rates. This method combines the weights of two previous methods by means of a test based on the positivity of the branch lengths estimated with the paralinear distance. ASAQ is statistically consistent when applied to data generated under the general Markov model, considers rate and base composition heterogeneity among lineages and does not assume stationarity nor time-reversibility. Second, we test and compare the performance of several quartet-based methods for phylogenetic tree reconstruction (namely QFM, wQFM, quartet puzzling, weight optimization and Willson’s method) in combination with several systems of weights, including ASAQ weights and other weights based on algebraic and semi-algebraic methods or on the paralinear distance. These tests are applied to both simulated and real data and support weight optimization with ASAQ weights as a reliable and successful reconstruction method that improves upon the accuracy of global methods (such as neighbor-joining or maximum likelihood) in the presence of long branches or on mixtures of distributions on trees.

Molecular phylogenetic reconstruction faces several problems, still nowadays. Even if one restricts to gene tree reconstruction, one has to take into account the amount of available data (which might be low with respect to the number of taxa), and depending on the method applied, the selection of a suitable evolutionary model, the inherent difficulty of estimating parameters for the most complex models, and the incorporation of heterogeneity across sites and/or across lineages, among others (cf. Jermiin et al. 2020; Zou et al. 2019, and the references therein).

Most of the available methods strongly depend on the evolutionary model assumed (this is the case of maximum likelihood, Bayesian or distance-based approaches, to a more or less extent), and some have to estimate the substitution parameters for each possible tree topology. Recent work minimizes the relevance of the selection of the substitution model if the tree topology is correctly inferred (see Abadi et al. 2019) and in this case, a *general Markov model* (GM for short) could be used (see also Kaehler et al. 2015). Regarding topology reconstruction, some methods that avoid parameter estimation and allow complex substitution models such as GM are those based on the *paralinear distance* or on *algebraic tools* (phylogenetic invariants and related tools, see Allman and Rhodes 2007).

The paralinear distance (Lake 1994) is a measure that attempts to estimate the evolutionary distance (in terms of expected number of substitutions per site) when sequences evolve under a GM model. It may overestimate the expected number of substitutions when the process is far from stationary, or branch lengths are long (see Kaehler et al. 2015; Zou et al. 2012), so more elaborate methods are provided in the quoted papers. However, it is a widely used distance due to its simple formula and its generality, and it has recently been proven to be consistent for the multispecies coalescent model in the ultrametric case as well, see Allman et al. (2021, 2019).

Several algebraic methods for phylogenetic reconstruction have been proposed in the last years, see, for example, SVDQuartets (Chifman and Kubatko 2014), Erik+2 (Fernández-Sánchez and Casanellas 2016)—both already implemented in PAUP* (Swofford 2003), Splitscores^1^ (Allman et al. 2016), or SAQ (Casanellas et al. 2021b). The methods Erik+2, Splitscores, and SAQ are based on the GM model and, in particular, they are not subject to stationarity or time-reversibility and account for different rates of substitution at different lineages (the so-called heterogeneity across lineages, see Appendix A). The three of them consider *algebraic* conditions in the form of rank constraints of a *flattening* matrix obtained from the observed distribution of characters on a sequence alignment. Only SAQ considers also the stochastic description of the evolutionary model (see Allman et al. 2014), which translates into *semi-algebraic* conditions. On the other hand, Erik+2 also allows across-sites heterogeneity as it is able to deal with data from a mixture of distributions on the same tree. Despite the potential of algebraic-based methods for topology reconstruction (already pointed out in the book by Felsenstein (2004)), some of these methods may not work well for short alignments especially in presence of the long branch attraction phenomenon. For instance, Erik+2 is highly successful on different types of quartet data (also on the Felsenstein zone) but requires at least one thousand sites to outperform maximum likelihood or neighbor-joining (briefly NJ) (Fernández-Sánchez and Casanellas 2016). By taking into account the stochasticity of the substitution parameters, SAQ overcomes this problem at the expense of performing slightly worse than Erik+2 for very large amounts of data (10,000 sites or more) (Casanellas et al. 2021b).

Algebraic methods are mainly aimed at recovering *quartet* topologies (or splits in some cases) and some account for the possibility of being implemented into *quartet-based methods*. Quartet-based methods (*Q-methods* for short) have been questioned in the literature. For instance, Ranwez and Gascuel (2001) evaluated two quartet-based methods, their weight optimization method (briefly WO) and the quartet puzzling method (QP) by Strimmer and von Haeseler (1996), and they weighted quartets using a maximum likelihood approach for the Kimura two-parameter model. Their main conclusion was that both QP and WO give worse results than neighbor-joining or than a maximum likelihood approach applied directly to the whole set of taxa. More recent methods such as QMC from Snir and Rao (2010), QFM Reaz et al. (2014), or wQFM from Mahbub et al. (2021) seem to be competitive with maximum likelihood and are scalable to large data sets. As pointed out by Ranwez and Gascuel (2001), the weaknesses of Q-methods are very likely due to the method of weighting the quartets rather than to the method of combining them.

As far as we are aware, the only works that evaluate the use of algebraic methods as input of quartet-based methods are Rusinko and Hipp (2012) (which is restricted to QP with the Jukes-Cantor model), Holland et al. (2012) (where squangles are applied to infer the quartets) and Chifman and Kubatko (2014) (for the coalescent model). On the other hand, the correct management of long-branch attraction is crucial for obtaining a successful quartet-based method (John et al. 2003). These claims and remarks motivate part of the present work. We expect that, as SAQ, Erik+2 and the paralinear method handle successfully the long-branch attraction problem, Q-methods with these weighting systems improve their performance. Moreover, Zou et al. (2019) proved that if a machine learning approach is applied to weighting the quartets, QP can have a similar performance than NJ especially under substitution processes that are heterogeneous across lineages. Precisely, handling heterogeneity of substitution rates across lineages is one of the key features of algebraic methods based on the GM model of nucleotide substitution.

The goal of this work is to test the performance of several quartet-based methods for phylogenetic tree topology reconstruction when applied with input weights from different methods consistent with the general Markov model. We test QFM, wQFM, QP, WO and the method WIL (proposed in Willson 1999) with different systems of input weights: two systems, PL and 4P (see Appendix A), derived from paralinear distances and the *four-point condition* (Lake 1994; Gascuel 1994; Mihaescu et al. 2009), and three systems based on algebraic and semi-algebraic methods, namely SAQ, Erik+2 and the new proposed method ASAQ (see below) that combines the paralinear distance and algebraic methods. We also provide a new implementation of QP, WO, WIL and the code for ASAQ. We test exhaustively all these combinations on simulated data evolving either under the GM model or the homogeneous general (continuous) time-reversible model (GTR) on twelve taxon trees, and also provide a comparison when input weights from a maximum-likelihood approach are used. We also compare the performance to a traditional NJ (from now on, *global NJ*) and a ML estimation of the tree (*global ML*) and test some of the methods on real data: the eight species of yeast studied in Rokas et al. (2003) and the Ratite mitochondrial DNA data studied in Phillips et al. (2009).

The new method ASAQ (standing for algebraic and semi-algebraic quartet reconstruction) is a topology reconstruction method for four taxa which combines Erik+2 and SAQ by means of the *paralinear method*. This is a quartet reconstruction method based on a statistic (see Eq. (1) in the Materials and Methods section) that assesses the positivity of the estimated length (using the paralinear distance) of the interior branch of the quartet. When data are unmistakably generated by a quartet, the topology output by any reconstruction method should be consistent with the positivity of this statistic. ASAQ uses the paralinear method to either ratify the results of Erik+2, or to rely on SAQ when there is an inconsistency between the outputs of Erik+2 and the paralinear method. By proceeding like this, ASAQ ensures an overall better performance on quartets than Erik+2, SAQ (and the paralinear method itself in most occasions).

As ASAQ is statistically consistent with the GM model, it can consider data evolving on a tree where base composition and instantaneous rate substitution patterns can differ among lineages (heterogeneity across lineages), and does not assume stationarity nor time-reversibility, see Appendix A. We test the performance of ASAQ on a wide scenario of simulated data: on the tree space proposed by Huelsenbeck (1995), on quartets with random branch lengths and on mixture data on the same topology with two categories. All simulations used data either generated from the GM model or from a GTR model, with different sequence lengths.

One can use ASAQ with mixtures of distributions on the same tree topology with two or three categories (or partitions) as this was already implemented by Erik+2. Although ASAQ is based on SAQ and the paralinear method, which are not guaranteed to be consistent on mixtures, it is also highly successful on this kind of data, see the Results section.

## Materials and Methods

In this section, we describe the new method ASAQ for quartet reconstruction, the quartet-based methods applied and the simulation studies performed.

### Description of the New Method ASAQ

ASAQ is a quartet reconstruction method based on a pair of previous methods by the authors (Fernández-Sánchez and Casanellas 2016; Casanellas et al. 2021b). For a phylogenetic tree *T* with an interior node *r* as root, we consider a Markov process on the alphabet $\{\texttt {A},\texttt {C},\texttt {G},\texttt {T}\}$ on *T* by assigning transition matrices $M_e$ at the edges of *T* and a distribution $\pi$ at *r*. As no restrictions are imposed on the transition matrices or $\pi$, this is usually called a *general Markov model* (GM) on *T*. One can compute the theoretical joint distribution *p* of patterns at the leaves of *T* in terms of the entries of $M_e$ and $\pi$ and we say that *p* *has arisen on* *T* with certain *substitution parameters*.

We consider fully resolved (unrooted) trees on a set of four taxa $[4]=\{1,2,3,4\}$: the three quartet trees shall be denoted as 12|34, 13|24, 14|23, according to the bipartition induced by the interior edge. Fernández-Sánchez and Casanellas (2016) introduced Erik+2, a reconstruction method essentially based on the rank of flattening matrices obtained from a distribution of nucleotides at the leaves of a tree. The method allows the possibility of dealing with data from mixtures of distributions with up to 3 categories (this upper bound is not computational, but rather a consequence of the theoretical basis of the method: distributions from mixtures with *c* categories satisfy that the rank of a certain $16 \times 16$-matrix is upper bounded by 4*c*, so in order to discriminate between such distributions and generic distributions we need $4c < 16$). In the more recent method SAQ, Casanellas et al. (2021b) create a more sophisticated method combining the rank of the flattening matrices with the stochastic information available in the data via a result of Allman et al. (2014). Both methods Erik+2 and SAQ, as well as their associated weighting system, are briefly described in Appendix A.1. They have been widely studied and the reader is referred to the cited publications for their performance on different scenarios.

As already explained in the introduction, while SAQ usually outperforms Erik+2 for short DNA sequence alignments (length $\le$ 1000), Erik+2 obtains better results as the length of the DNA alignment increases. This consideration leads us to introduce ASAQ, a new combined method of Erik+2 and SAQ that tries to apply one or the other according to whether the input pattern distribution is consistent with the positivity of the estimated length of the interior branch. To this end we use the paralinear distance and the paralinear method (see Appendix A.1, Lemma A.1 and Lake (1994)).

For a distribution of patterns at the set of leaves, $p\in {\mathbb {R}}^{256}$, we compute all paralinear distances $d_{x,y}$ between pairs $x,y \in [4]$. Then, given a bipartition *A*|*B* of the set [4], $A=\{i,j\}$, $B=\{k,l\}$, we define the following quantity

1 $$\begin{aligned} {p\ell }_{A|B}(p)=\min \{d_{i,k}+d_{j,l} \,, \, d_{i,l}+d_{j,k}\}-d_{i,j}-d_{k,l}. \end{aligned}$$

The quantity above is the “neighborliness” measure used in Gascuel (1994), the “paralinear method” used in Lake (1994), and was presented by Buneman (1971) as a measure of twice the length at the interior edge (when *d* is a tree metric). It is worth pointing out that at most one of the three values ${p\ell }_{12|34}(p)$, ${p\ell }_{13|24}(p)$ and ${p\ell }_{14|23}(p)$ is strictly positive if all paralinear distances above are non-negative, see Lemma A.2. We denote by $\texttt {PL} (p)$ the collection of these values:

$$\begin{aligned} \texttt {PL} (p)=({p\ell }_{12|34}(p), {p\ell }_{13|24}(p), {p\ell }_{14|23}(p)). \end{aligned}$$

If *p* has arisen on a quartet *A*|*B* and the entries of the Markov matrix at the interior edge were strictly positive, both quantities inside the minimum in Eq. (1) are equal (as can be deduced from the 4-point condition). Moreover, ${p\ell }_{A|B}(p)$ is the unique positive quantity in the triplet $\texttt {PL} (p)$ and its value coincides with twice the paralinear distance of the interior edge (see Theorem A.3). The *paralinear method* is the quartet reconstruction method that outputs the tree *T* with highest ${p\ell }_{T}$ value. It is a statistically consistent quartet-inference method (Theorem A.3).

Therefore, we propose the following quartet reconstruction method ASAQ: it checks whether both methods Erik+2 and the paralinear method PL output the same quartet, and

1. if Erik+2 and PL agree, then ASAQ outputs the topology and weights of Erik+2;
2. if they do not agree or some paralinear distances $d_{x,y}$ are negative, then ASAQ outputs the topology and weights of SAQ.

An inconsistency between Erik+2 and the paralinear method implies that data are far from having arisen on a tree with *stochastic* parameters (note the role of the positivity of the entries of the Markov matrix in Lemmas A.1 and A.2). The positivity of transition matrices implies semi-algebraic conditions on the joint distributions at the leaves (Allman et al. 2014) and in this case we rely on SAQ, as it is the unique method that takes into account both these semi-algebraic conditions and the algebraic constraints considered by Erik+2. In Theorem A.3 we prove that ASAQ is as well a statistically consistent quartet reconstruction method for the general Markov model and in Table 5 we show the percentage of discordance between PL and Erik+2, and the success of SAQ in these cases.

We cannot claim that ASAQ is statistically consistent for mixtures because consistency is not known to hold for the paralinear method or SAQ in this scenario. However, the simulation studies in Casanellas et al. (2021a) show a good performance of SAQ in mixture data from the same tree and this will lead to a good performance of ASAQ as well (see the Results section). We denote by ASAQ ($m=k$) the use of ASAQ with Erik+2 estimating mixtures on the same tree with *k* categories. The limit on the number of categories $m=3$ for quartets comes from the theoretical foundations of Erik+2, as a larger amount of categories would make unfeasible the identifiability of the tree topology by this method.

As a topology reconstruction method, the paralinear method is highly successful (see Results section, Fig. 2). Nevertheless, we found that using the paralinear method in order to ratify or not the results of Erik+2 gives a better performance on topology reconstruction.

### Quartet-Based Methods (Q-Methods)

We have implemented different quartet-based methods (Q-methods) with different input weights. All of them seek for a tree that maximizes weighted quartet consistency. Quartet puzzling (QP), weight optimization (WO) and Willson’s (WIL) methods have been programmed in C!++! and wQFM has been used with the implementation provided in Mahbub et al. (2021). QP amalgamates quartets in a randomized order and seeks to maximize the total sum of weights; we provide a new implementation of QP different from the one in Schmidt et al. (2002) since we wish to apply the method with systems of weights not based on likelihoods. Weight optimization uses quartet weights to dynamically define the taxon addition order, seeking to maximize the total weight at each step. WO is known to reconstruct the correct tree if the input quartets are correctly weighted (i.e. if all quartets of the original tree have the highest weight among the tree possible weights of the corresponding 4-tuple), see Ranwez and Gascuel (2001). Instead of constructing a tree that maximizes the total weight at each step, the essential idea of Willson’s method is attaching new taxa in such a way that the new tree at each step is highly consistent with the input quartets. QP, WO and WIL are initialized at a random 4-tuple. Since the output of these Q-methods strongly depends on the choice of the initial quartet, each one of them has been applied 100 times to each alignment and then the majority rule consensus tree (briefly MRCT) of these 100 replicates has been computed.

On the contrary, wQFM amalgamates quartets following a divide and conquer approach and implicitly gives the same importance to all quartets (and does not depend on an initial quartet choice). Although wQFM was specially designed to build species trees from gene trees, it can also consider other input weights. We use wQFM with quartets weighted by different methods, but also with unweighted quartets. In this last case we refer to the method as QFM Reaz et al. (2014) and the weights are transformed to 1 for the quartet output by the method and to 0 for the other two quartets.

In order to evaluate the difference between two trees, we use the Robinson–Foulds distance (RF for short), (Robinson and Foulds 1981). For the computation of the majority rule consensus tree and the RF distance, the available functions in the Python Library *DendroPy* have been used, see Sukumaran and Holder (2010).

**Input Weights**

We require the weights of the quartet reconstruction methods to be positive and normalized. Details about the input weights obtained from ASAQ are provided in the previous section. Further details about the weighting system for all the considered methods are moved to Appendix A.1. For the paralinear method the weights are denoted as PL and are obtained after normalizing the exponentials of the scores given by Eq. (1); 4P is a slight modification of this method, see Appendix A.1. We consider already published weighting systems for SAQ, Erik+2 and maximum likelihood (ML): see Fernández-Sánchez and Casanellas (2016) for Erik+2, Casanellas et al. (2021b) for SAQ, and the posterior probabilities used in Strimmer et al. (1997) for ML.

Maximum likelihood weights (which will be denoted as ML) have been computed assuming the most general continuous-time homogeneous model (same instantaneous rate matrix throughout the tree but no constraints on the entries of the rate matrix or assumption of stationarity); this is the Lie Markov model listed as 12.12 in Fernández-Sánchez et al. (2015) and was denoted as ML(homGMc) in Fernández-Sánchez and Casanellas (2016). To this aim we used the *baseml* program from the *PAML* library (Yang 1997) with the UNREST model and let it infer the instantaneous rate matrix (common to all lineages) and the distribution at the root. Note that in order to use weights obtained from ML, we need to obtain the estimates of the maximum likelihood for the three possible quartets. This is unfeasible when the method does not converge (which often happens for the quartets which did not generate the data).

### Description of the Simulated Data

We consider two different scenarios of simulated data: one for testing ASAQ as a quartet reconstruction method (described in Sect. 1.3.1) and another for testing different Q-methods with several weighting systems (see Sect. 1.3.2). For the first we use the simulated data introduced by Fernández-Sánchez and Casanellas (2016), whereas for the second we follow the approach of Ranwez and Gascuel (2001) and consider 12-leaf trees.

**Evolutionary models** For the trees described below, we generate data evolving either on a general Markov model (GM, see Sect. 2.1) or on a homogeneous general time-reversible model (GTR). By a homogeneous GTR model we mean a continuous-time GTR model that shares the same instantaneous mutation rate matrix *Q* across all branches of the tree. GTR data have been generated using Seq-gen (Rambaut and Grass 1997), while GM data have been generated using the software GenNon-h (Kedzierska and Casanellas 2012) available at https://github.com/Algebraicphylogenetics/GenNon-H. For GTR data we specified in each setting a stationary distribution and a rate matrix common to all generated trees (see below for details on the chosen rates in each case), whereas for GM data the transition matrices are randomly generated for each alignment (according to the specified branch lengths).

#### Simulated Data for Quartet Reconstruction

**Tree space** The first data set we use to test ASAQ corresponds to the tree space suggested by Huelsenbeck (1995). We consider quartets as in Fig. 9a in Appendix B, with branch lengths given by a pair of parameters *a* and *b* which vary between 0 and 1.5 in steps of 0.02. Branch lengths are always measured as the expected number of elapsed substitutions per site. The resulting *tree space* is shown in Fig. 9b in Appendix B. The upper left region of this tree space corresponds to the “Felsenstein zone”, which contains trees subject to the long branch attraction phenomenon. For each of the two nucleotide substitution models considered in the paper, GM and GTR, and for each pair (*a*, *b*) of branch lengths, we have simulated one hundred alignments. The rates for GTR data were chosen as in Fernández-Sánchez and Casanellas (2016), subsection Description of the Data, p.284. The considered alignment lengths are of 500, 1 000 and 10 000 sites.

**Random branch lengths** Following Casanellas et al. (2021b), we test ASAQ on 10 000 alignments generated from quartets whose branch lengths are randomly generated according to a uniform distribution in the intervals (0, 1) or (0, 3). These alignments are obtained according to both substitution models, GM and GTR, and are either 1 000 or 10 000 sites long. We represent the weights output by ASAQ in a ternary plot (also called a simplex plot) as in Strimmer and von Haeseler (1997).

**Mixture data** The performance of ASAQ is also tested in the scenario of data sampled from a mixture of distributions. According to the approach of Kolaczkowski and Thornton (2004), we consider the mixture of distributions as follows. We partition the alignment into two categories of the same sample size both evolving under the GM model on the same quartet topology as Fig. 9a but the first category corresponds to branch lengths $a = 0.05$, $b = 0.75$, while the second corresponds to $a = 0.75$ and $b = 0.05$ (see Fig. 10 in the Appendix). The internal branch length takes the same value in both categories and varies from 0.01 to 0.4 in steps of 0.05. The total length of the alignments considered is 1 000 or 10 000 sites.

**Fig. 1:**
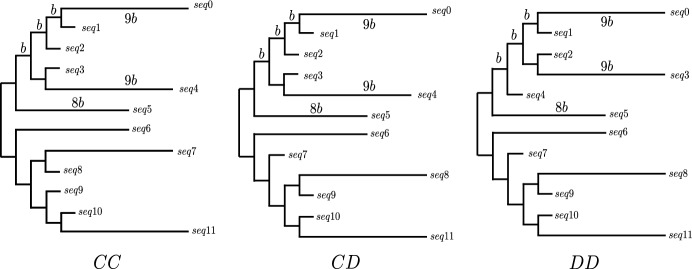
The three different tree topologies *CC*, *CD* and *DD* on 12 taxa considered to test the Q-methods with different weighting systems. They are obtained by glueing a combination of two trees (*C* and *D*) by the root. Here, the parameter *b* represents the length of internal branches. These tree topologies have been taken from (Ranwez and Gascuel 2001)

#### Simulated Data for Larger Trees

We followed Ranwez and Gascuel (2001) to test the performance of different quartet-based methods (QP, WO and WIL) with different weighting systems. To this end, we considered the three 12-taxon topologies depicted in Fig. 1 denoted as *CC*, *CD* and *DD*, and fixed the ratio among branch lengths as in Ranwez and Gascuel (2001), depending on a parameter *b* denoting the internal branch length, which is varied in the set $\{0.005,0.015,0.05,0.1,0.25,0.5\}$.

For each tree topology and for each *b*, we have considered 100 alignments with lengths 600 (in order to match the alignment length considered in Ranwez and Gascuel (2001)), 5 000, and 10 000 generated under the GM model. For *DD* topology, we have also generated data under the GTR model with $b=0.005,\ldots , 0.1$, and instantaneous rates 2 (A$\rightarrow$C), 5 (A$\rightarrow$G), 3 (A$\rightarrow$T), 4 (C$\rightarrow$G), 1 (C$\rightarrow$T), 2 (G$\rightarrow$T) and equal base frequency (we chose *DD* because it is arguably the hardest to reconstruct, see the Results section).

**Mixture data** We have considered a 2-category mixture model, that is, we generated alignments evolving on a the topology CD but whose sites evolve following two systems of substitution parameters: the first system corresponds to the branch lengths described in the tree *CD*, while the second system corresponds to *CD* after exchanging the branch lengths of *seq*3 and *seq*4, and the lengths of *seq*7 and *seq*8 (see Fig. 11 for details). The parameters that were varied in this framework were the proportion *p* of sites in the first category (which was varied in 0.25, 0.50,  and 0.75) and the internal edge length *b* which was varied as above in 0.005, 0.015, 0.05 and 0.1. The lengths of the alignments considered were 600, 1 000 and 10 000 bp.

### Real Data

**Yeast data** We analyze the performance of ASAQ on real data on the eight species of yeast studied in Rokas et al. (2003) with the alignment consisted of the concatenation of 42 337 s codon positions of 106 genes as provided by Jayaswal et al. (2014). We investigate whether the quartets output by ASAQ support the tree *T* of Rokas et al. (2003), the alternative tree $T'$ of Phillips et al. (2004) (see Fig. 12), or the mixture model proposed by Jayaswal et al. (2014). Although the tree *T* is widely accepted by the community of biologists, its correct inference is known to depend on the correct management of heterogeneity across lineages, as an inaccurate underlying model usually reconstructs $T'$ (Rokas et al. 2003; Phillips et al. 2004; Jayaswal et al. 2014). According to Jayaswal et al. (2014), these data are best modeled by considering, apart from heterogeneity across lineages, two different rate categories (discrete $\Gamma$ distribution) plus invariable sites. In our setting, this is translated into a mixture distribution with 3 categories ($m=3$ in ASAQ).

**Ratites and tinamous mitochondrial data** The phylogeny of ratites and tinamous has been debated for some time (see Phillips et al. 2009, and the references therein) and is still controversial (Benito et al. 2022). There is evidence of a higher rate of evolution among the tinamous relative to the ratites (see, e.g., Paton et al. 2002), so these data are appropriate for analysis by the methods proposed here. Moreover, the recoding used in Phillips et al. (2009) to sort out this problem is questioned in Vera-Ruiz et al. (2021). We do our analyses using a 3506 sites alignment consisting of the third codon position of the mitochondrial DNA coding alignment for 24 DNA sequences provided in this last paper. We run WO and QP with weights obtained by ASAQ, SAQ, Erik+2, 4P, PL. For each combination of methods we used all quartets but also a random selection of a subset of input quartets of size either one hundred, 2125 (approximately 20% of all possible quartets), or 5313 (equal to 50%), and then we performed the MRCTs. This was aimed at testing the sensitivity of the method to the amount of input data (comparing the performance when only a portion of the quartets is used) in terms of scalability of the methods.

We analyze the results obtained in comparison to the following trees: (A) the tree that groups Tinamous and Moas as proposed by Phillips et al. (2009) and displayed in Vera-Ruiz et al. (2021), Fig. 4B the consensus tree of Fig. 1a of Phillips et al. (2009) and (C) the Ratite paraphyly tree of Fig. 1b of Phillips et al. (2009). If we call CEK to the largely established clade CEK=((cassowary, emu), kiwis), then these trees can be summarized as:

A: (outgroup, neognathus, (ostrich, (rheas, ((moas, tinamous), CEK))));
B: (outgroup, neognathus, (tinamous, (moas, rheas, (ostrich, CEK))));
C: (outgroup, neognathus, ((tinamous, moas), (rheas, (ostrich, CEK))))  ,

where the outgroup comprises sequences of alligator and caiman (see Fig. 7 for a description of the species involved). As unrooted trees, A and C have 21 interior edges and B has 20.

## Results

In this section, we describe the results obtained and benchmark them with published results for the sake of completeness. The interested reader is referred to the corresponding papers for details of the methods therein.

### Results on Quartets

#### Tree Space

**Fig. 2:**
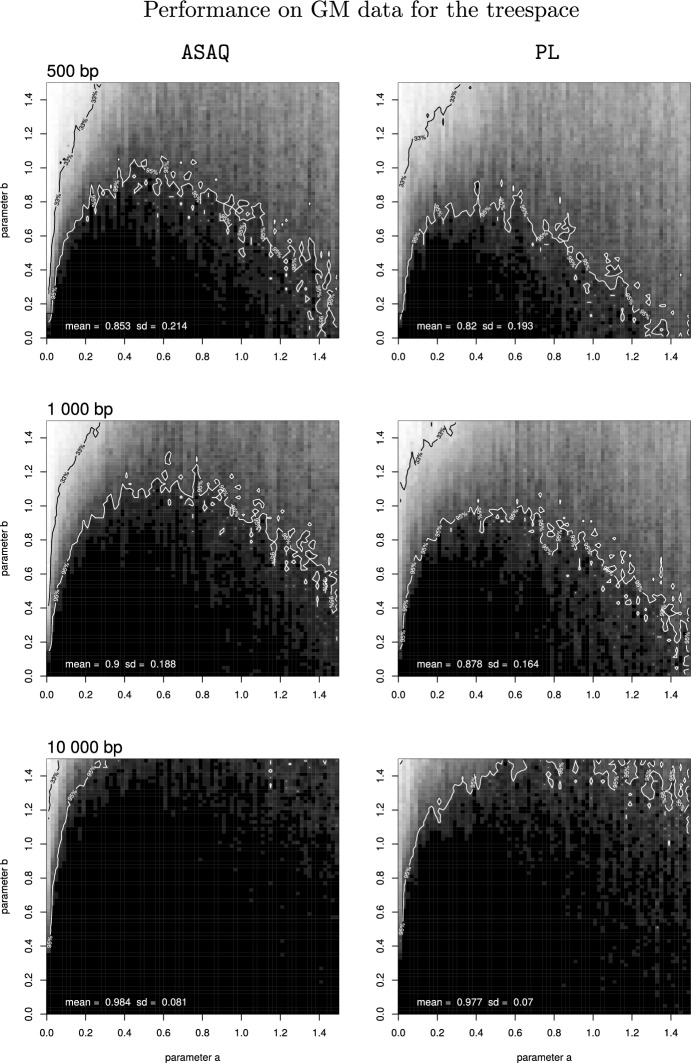
Performance of ASAQ (left) and PL (right) on the tree space of Fig. 9b on alignments of length 500 bp (top), 1 000 bp (middle) and 10 000 bp (bottom) generated under the GM model. Black is used to represent 100% of successful quartet reconstruction, white to represent 0%, and different tones of gray the intermediate frequencies. The 95% contour line is drawn in white, whereas the 33% contour line is drawn in black

The performance of ASAQ and PL on data generated on the tree space of the previous section (*Materials and Methods*) is represented in Fig. 2 (for GM data) and in Fig. 13 in the Appendix (for GTR data). In black we represent 100% success, in white 0% success, and gray tones correspond to regions of intermediate success accordingly. The 95 % and 33 % isoclines are represented with a white and black line, respectively. These simulation studies show a consistent performance according to the results by Huelsenbeck (1995) and Fernández-Sánchez and Casanellas (2016), with the usual decreasing performance at the Felsenstein zone and an improvement of both methods with sample size. Figures 2 and 13 show that the performance of ASAQ is better than that of PL, for both GM and GTR data.

For completeness, the average performance of ASAQ and PL on this tree space for different alignment lengths and underlying models is compared to other methods in Table 1: we include the average results of SAQ (Casanellas et al. 2021b, as shown in) and Erik+2 and ML as published in Fernández-Sánchez and Casanellas (2016). This comparison shows that for GM data the best results are achieved by ASAQ, while for GTR data ML obtains the best performance for alignments of length 500 and 1 000 bp and Erik+2 does so for long alignments (10 000 bp).

**Table 1:** Average success of several methods applied to data simulated on the tree space of Fig. 9b). ASAQ and PL are compared to the results for SAQ obtained in (Casanellas et al. 2021b), and for Erik+2 and maximum likelihood ML in (Fernández-Sánchez and Casanellas 2016). MLestimates the most general continuous-time homogeneous process 12.12 when data are generated under a GM model, while ML estimates a homogeneous GTR model when data are generated under GTR, see (Fernández-Sánchez and Casanellas 2016, Table 1). In each row of the table, the highest success is indicated in bold font

Simulations	Base pairs	ASAQ	SAQ	Erik+2	PL	ML
Average success of different methods on the tree space						
GM	500	**85**.**3**	84.6	72.4	82.1	72.1
	1 000	**90**	88.8	80.3	87.8	73.6
	10 000	**98**.**4**	96.8	97.1	97.7	75.4
GTR	500	79.9	78.4	74.8	78.7	**88**.**0**
	1 000	86.9	83.5	84.3	85.8	**93**.**4**
	10 000	97.9	94.5	**99**.**2**	96.9	98

#### Random Branch Lengths

**Fig. 3:**
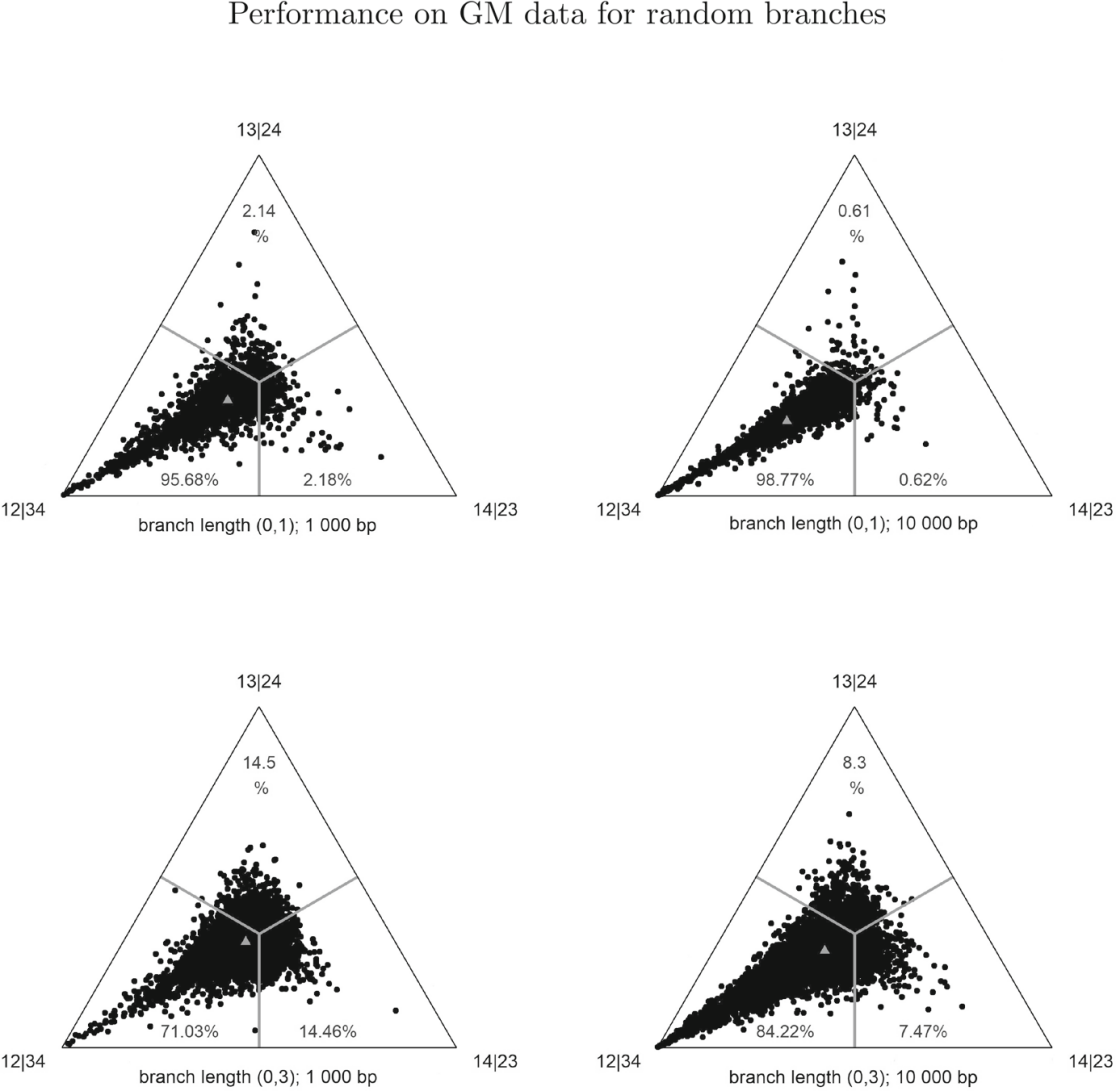
Ternary plots corresponding to the weights of ASAQ applied to 10000 alignments generated under the GM model on the 12|34 tree. On each triangle the bottom-left vertex represents the underlying tree 12|34, the bottom-right vertex is the tree 13|24 and the top vertex is 14|23. The small gray triangle depicted represents the average point of all the dots in the figure. Top: correspond to trees with random branch lengths uniformly distributed between 0 and 1; bottom: random branch lengths uniformly distributed between 0 and 3. Left: 1 000 bp; Right: 10 000 bp. The analogous ternary plots for SAQ can be found in Garrote-López (2021)

To visualize the overall distribution of the weights of ASAQ applied to trees with random branch lengths, in Fig. 3 we show ternary plots corresponding to the GM alignments described in Sect. 1.3.1. The ternary plots of the performance of ASAQ when applied to the same setting with data generated under the GTR model are shown in Fig. 14. In Table 2 we display the summary of average success of ASAQ on these data.

Note that Table 2 shows a high performance of the method and the ternary plots show a clear distribution of points towards the bottom left corner (which represents the correct quartet) and weights symmetrically distributed on the other corners. In particular, the method is not biased towards any of the incorrect topologies. We note that the level of success exhibited is quite sensitive to the branch length, being much higher for branch lengths in (0,1) than in (0,3). We do not appreciate a remarkable difference between the performance of ASAQ when applied to GTR data.

**Table 2:** Average success of ASAQ on alignments of lengths 1 000 and 10 000 bp generated on the tree 12|34 under the GM and GTR models with random branch lengths uniformly distributed in (0,1) (first row) and (0,3) (second row). The plots corresponding to these data are shown in Figs. 3 and 14

Branch length	GM		GTR	
	1 000 bp	10 000 bp	1 000 bp	10 000 bp
Average success of ASAQ applied to data generated on 12|34 with random branch lengths				
(0,1)	95.68	98.77	94.65	98.42
(0,3)	71.03	84.22	69.37	85.12

#### Mixture Data

**Fig. 4:**
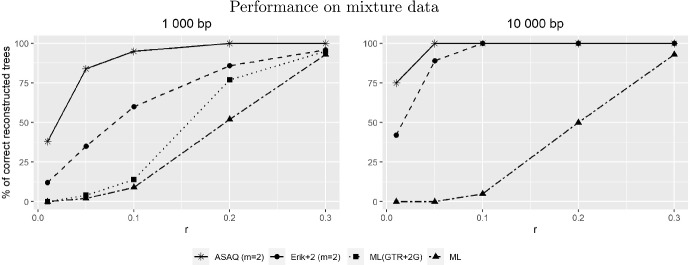
These plots represent the percentage of correctly reconstructed trees by several methods applied to the mixture data described in the Method section; the value *r* refers to the branch length of the interior edge of the trees (Fig. 10). We compare the results of ASAQ ($m=2$) and the results presented in (Fernández-Sánchez and Casanellas 2016, Figure 5) for Erik+2 ($m=2$), ML  (as usual on the 12.12 model), and a ML estimating a heterogeneous across lineages GTR with two categories of discrete $\gamma$ rates across sites denoted as ML(GTR+2$\Gamma$) (only for length 1000 bp)

In Fig. 4 we show the performance of the method ASAQ with $m=2$ categories when applied to data from mixtures as described in Sect. 1.3.1 and in Fig. 10. Based on the results of Fig. 5 in Fernández-Sánchez and Casanellas (2016), we also provide a comparison to the success of Erik+2 $(m=2)$ and two versions of maximum likelihood on the same data. We did not include the performance of SAQ, as it is very similar to that of ASAQ.

Figure 4 shows an increasing accuracy of all methods when the value of the parameter *r* (the branch length of the interior edge of the two trees involved) is increased. This was expected as larger values of *r* represent larger divergence between sequences at the left and the right of the trees. We note that ASAQ ($m=2$) outperforms the other methods, with a high level of success in all cases, even when the length of the alignments is 1 000 bp. The average success of ASAQ $(m=2)$ applied to the simulated data is $96.64\%$ for 1 000 bp, $99\%$ for 10 000 bp and $99.92\%$ for 100 000 bp (results not shown).

### Results of Q-Methods

We proceed to describe the performance of wQFM, QFM, WO, WIL and QP (see the subsection on Quartet-based methods ) applied to the input weights from ASAQ, SAQ, Erik+2, PL, 4P and ML on the trees *CC*, *CD* and *DD* presented in Sect. 1.3.2. The weights from PL and 4P are both defined in terms of the paralinear distance (see Appendix A.1), and they produce similar results. Because of this, we omit the performance of 4P in some cases. We also add the results obtained from a global NJ applied to the same data (using the NJ algorithm implemented in the R package APE of Paradis et al. (2004) with paralinear distance) and also global ML using IQ-TREE 2 (Minh et al. 2020) with the most continuous-time homogeneous general model 12.12.

#### Unmixed Data

**Fig. 5:**
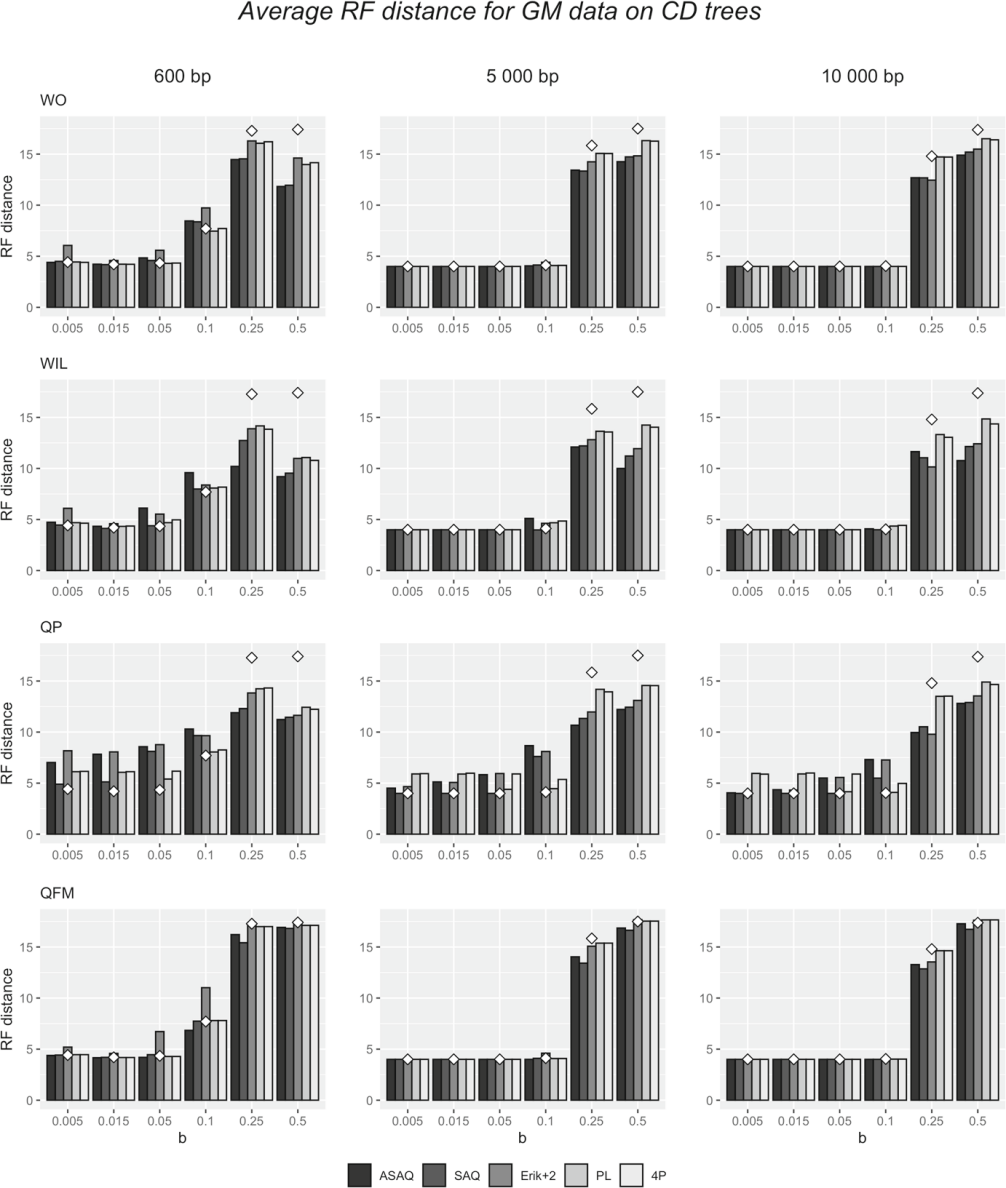
Average Robinson–Foulds distance for GM data simulated on the tree *CD* with alignment length 600 bp (left), 5 000 bp (center) and 10 000 bp (right). The Q-methods WO (first row), WIL (second row), QP (third row), and QFM (last row) are applied with different systems of weights, namely ASAQ, SAQ, Erik+2, PL and 4P. The white diamonds represent the average RF distance of the tree reconstructed using a global NJ with paralinear distances. Specific values of these results are detailed in Tables 6 and 10, and results with ML weights are included in Table 9

The results on simulated GM data obtained by the combination of methods and weights presented in this paper are summarized in Fig. 5 for *CD*, in Fig. 15 for *CC* and in Fig. 15 for *DD*. The results for GTR data (for the tree *DD*) are shown in Fig. 17. The height of the bars shows the average of the RF distance from the original tree to the consensus tree of 100 replicates for each of the 100 generated alignments. The values of these results are detailed in Tables 6 (for *CC*), C.3 (for *CD*) and C.4 (for *DD*) for GM data and in C.7 for GTR data on *DD*. For comparison, the results of a global NJ and global ML (which has a similar performance as a global NJ) applied to these data is displayed in Table 10 (see also the results of the global NJ in Figs. 5, 15 and 16). In Table 9, we show the results obtained when applying ML weights. Since ML did not converge for some quartets (especially in the presence of long branches and when trying to maximize the likelihood for GM data generated on another quartet), we write between parentheses the number of alignments considered for ML (in the computation of the average RF distance we neglected the alignments where ML did not converge).

By comparing the performance of the four Q-methods, we observe a slightly better performance of wQFM, QFM WIL and WO compared to QP. The accuracy drops for long branches in all methods, but wQFM and WO seem to perform slightly better for short and medium branches ($b\le 0.1$) while WIL does better for longer branches $b\ge 0.25$. Among all Q-methods, QP is the most sensitive to the choice of the system of weights.

Another general remark is that all Q-methods with their best system of weights outperform a global NJ and a global ML for $b>0.1$ on GM data, while none of them (with any system of weights) beats a global NJ or ML for *b* smaller than or equal to 0.1. Figure 17 shows that a global NJ is the best option for GTR data on *DD* trees.

All reconstruction algorithms have had considerably more success when reconstructing the tree *CC* in contrast to *CD* or *DD* for GM data, in concordance with the results obtained by Ranwez and Gascuel (2001). In the tree topologies *CD* and *DD*, the distance between *seq*9 and *seq*10 is 4*b*, while the distance between *seq*8 and *seq*11 is 20*b*. The same happens between species *seq*1 and *seq*2, and *seq*0 and *seq*3 of the *DD* topology. Thus, for an alignment generated from these trees, there is a high probability that two separate lineages evolve in a convergent manner to the same nucleotide at the same site, creating a *long branch attraction* situation and making the reconstruction methods to infer the wrong topology. wQFM, QFM, WO and WIL are especially successful when dealing with *CC* and *CD* trees (Fig. 15 and Fig. 5) and short branches, probably because these Q-methods succeed in reconstructing the *C* subtree.

Weights from ASAQ, SAQ, Erik+2, PL and 4P produce overall comparable results, although ASAQ and SAQ do better when dealing with long branches. wQFM with seems to work slightly better with quartets weighted by SAQ, PL and 4P over Erik+2 or ASAQ (although it does not perform as well as WO when dealing with long branches). On the contrary, in the unweighted version, ASAQ seems to provide the best input quartets for QFM (note that PL and 4P provide the same input for QFM, so they are displayed together in the tables). We also note an improvement in the results when the length of the alignment increases, especially for the *CC* case or for GTR data (Fig. 17) and hardly noticeable for the other two trees on GM data.

The simulation study shows a big difference between the general performance of the Q-methods with input weights from ASAQ, Erik+2 and PL in contrast to weights obtained by ML, especially when reconstructing the *CC* and *CD* trees (compare Tables 6, 7, 8 and 9). This could probably be due to the inconsistency between the ML weights computed and the general Markov model used to generate the data (see subsections *Quartet-based methods* and *Description of the simulated data*). Even when the model used for ML estimation matches the model that generated the data (as for GTR, see Table 11), ML seems to be the worse weighting system. When data are generated under the GTR model on *DD* trees (Fig. 17 and Table 11), all Q-methods (except for ML weights) have a remarkable high performance when alignments are long enough ($\ge$ 5 000 bp), especially WO. For short alignments, PL seems a good choice for the input weights with these data. All in all, we observe that both ASAQ and PL weights (combined with one or another Q-method) give rise to good results (much better than ML weights) comparable with the results obtained by a global NJ or ML, and even better when dealing with long branches.

#### Mixture Data

**Fig. 6:**
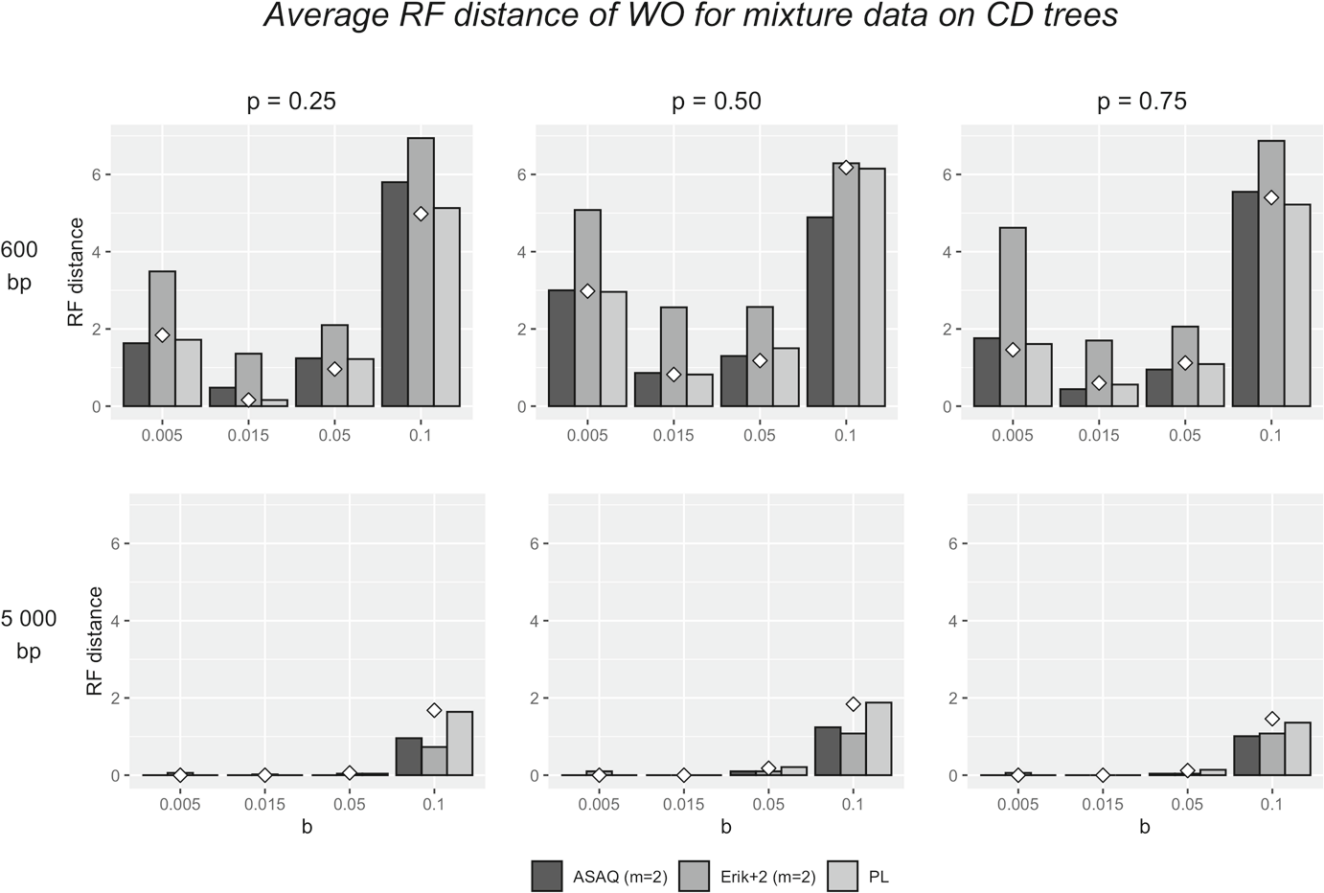
Average Robinson–Foulds distance on mixture data simulated on the tree *CD* for $p=0.25$ (left), $p=0.5$ (middle) and $p=0.75$ (right) with alignment length 600 bp (above) and 5 000 bp (below). We omit the results for 10 000 bp as they are very similar to those obtained for 5 000 bp. The Q-method WO is applied with different systems of weights, namely ASAQ with 2 categories, Erik+2 with 2 categories, and PL. The white diamond represents the result of a global NJ with the paralinear distance. Results obtained by WO, QP, WIL for the different proportions between the two categories, $p=0.25$, $p=0.5$ and $p=0.75$, can be found in Tables 12, 13 and 14, respectively. These tables also include results with ML weights

The results on mixture data obtained by a global NJ and by QP, WO, and WIL with input weights from PL, ASAQ and Erik+2 adapted to 2-category data (as described in Sect. 1.3.2) are summarized in Fig. 6 for WO and in Tables 12, 13 and 14 for all methods. Tables 12, 13 and 14 have a similar structure as Tables 6, 7 and 8 and correspond to the results obtained for different proportions between the two categories; $p=0.25$, $p=0.5$ and $p=0.75$, respectively. (We recall that the proportion *p* of sites of the first category of the alignment were generated assuming the branch lengths of the *CD* tree in Fig. 1.) It is worth pointing out that the reconstruction of mixture data from *CD* trees is more accurate on average than when applied to unmixed data (Fig. 5). This is probably due to the fact that when the branch lengths of species *seq*3, *seq*4 and *seq*7, *seq*8 are exchanged (Fig. 1), reconstruction methods have an easier job to make the appropriate splits, since the long branch attraction situation that was provoked by the quartet of species $\{seq8,seq9,seq10,seq11\}$ does no longer exist with the new branch lengths. This is consistent with the observation that the reconstruction results are more accurate for low values of *b*. For short alignments (600 bp), we note that ASAQ and PL weights provide better results than Erik+2. For 5 000 bp ASAQ and Erik+2 outperform PL. Moreover, if $b=0.1$, the combination WO+ASAQ or WO+Erik+2 beats a global NJ. For 10 000 bp, the difference between performance is even larger (see Tables Tables 12, 13 and 14). As expected, the length of the alignment improves the performance of these methods, reducing the impact of the long branch attraction effect for high values of *b*.

### Results on Real Data

#### Yeast Data

As shown in Table 3, the claim by Jayaswal et al. (2014) is corroborated by our analysis: by performing a MRCT on 100 random initial quartets, WO+ASAQ reconstructs *T* when 3 categories are considered, but it reconstructs $T'$ otherwise. Similarly, the RF distance of the MRCT obtained by WIL+ASAQ to the tree *T* is smaller than the RF distance to $T'$ only when 3 categories are considered. When applied to these data, NJ with paralinear distances reconstructs the tree *T*.

It should also be mentioned that several studies have analyzed this data set seeking to build a species network from individual gene trees. These works mostly support a hybridization event involving *S. kudriavzevii* and *S. bayanus*, resulting in a species network with at least one 4-cycle that could give rise to the inconsistencies between trees *T* and the $T'$; see for instance Allman et al. (2019); Holland et al. (2004) and Yu et al. (2011).

**Table 3:** Robinson–Foulds distance of the consensus tree obtained by WIL and WO with ASAQ weights (with different number of categories) applied to the trees *T* and $T'$ suggested in Rokas et al. (2003) and Phillips et al. (2004), respectively

	RF distance to *T*		RF distance to $T'$	
	WIL	WO	WIL	WO
ASAQ (m=1)	1.974	2	0.026	0
ASAQ (m=2)	1.922	2	0.078	0
ASAQ (m=3)	0.004	0	1.996	2

#### Ratites and Tinamous

First, we analyze the results obtained by 50 majority rule consensus trees obtained from WO and QP on 100 input quartets weighted with different methods. In Table 4 we show the average results in comparison to the trees *A*, *B* and *C* detailed in Sect. 1.4. The number of interior edges obtained by each method shows that WO produces more resolved trees than QP (independently of the weighting method) and that ASAQ, SAQ and Erik+2 (m=1) give rise to more resolved trees than PL and 4P with both WO and QP.

**Table 4:** For each weighting system (indicated in the left column) combined with either QP or WO, the first column depicts the average number of interior edges of 50 MRCTs on Ratites real data. The average RF distance and *normalized* RF distance (that is, dividing by the number of interior edges) from the MRCTs to the trees A, B and C (see Sect. 1.4) is shown in the remaining columns. The last row shows the RF distance and the normalized RF distance from the global NJ tree (obtained using paralinear distances) to trees A, B and C

			Tree A				Tree B				Tree C			
	Interior edges		RF dist		Norm. RF		RF dist		Norm. RF		RF dist		Norm. RF	
Weights	QP	WO	QP	WO	QP	WO	QP	WO	QP	WO	QP	WO	QP	WO
SAQ	14.3	20.5	9.7	10.0	0.3	0.2	6.7	7.0	0.2	0.2	9.7	10.0	0.3	0.2
ASAQ $_{m=1}$	13.8	19.5	9.7	11.9	0.3	0.3	6.7	8.9	0.2	0.2	9.7	11.9	0.3	0.3
ASAQ $_{m=2}$	14.0	19.8	9.0	9.9	0.3	0.2	6.2	6.9	0.2	0.2	9.0	9.9	0.3	0.2
Erik+2 $_{m=1}$	11.0	19.0	12.3	14.4	0.4	0.4	11.3	12.0	0.4	0.3	12.3	14.4	0.4	0.4
Erik+2 $_{m=2}$	10.1	15.8	12.0	11.8	0.4	0.3	11.0	9.4	0.4	0.3	12.0	11.8	0.4	0.3
PL	8.5	12.4	15.3	17.4	0.5	0.5	14.3	16.4	0.5	0.5	15.3	17.4	0.5	0.5
4P	8.3	12.7	15.3	17.7	0.5	0.5	14.3	16.7	0.5	0.5	15.3	17.7	0.5	0.5
NJ	21		8		0.19		5		0.12		8		0.19	

**Fig. 7:**
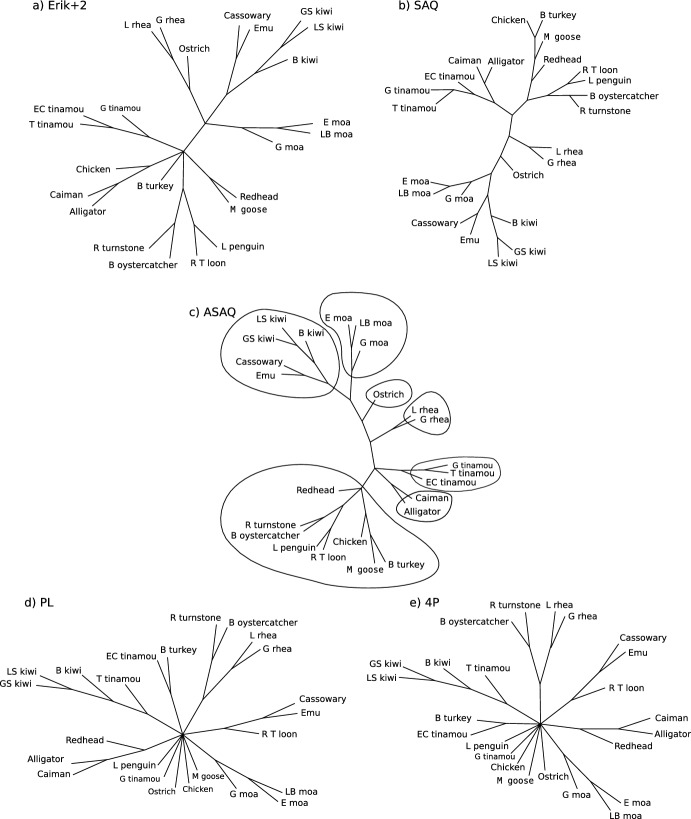
MRCTs obtained for ratites and tinamous data (see *Materials and Methods* in the main document ) by WO applied to 2125 initial quartets with weights from **a** Erik+2 ($m=1$), **b** SAQ, **c** ASAQ ($m=1$), **d** PL and **e** 4P. The trees obtained with these weights from 5313 initial quartets or with both $m=1$ and $m=2$ are the same as shown. All edges are shown with the same length, and they do not represent evolutionary distance. Tree c) (ASAQ) corresponds to the phylogeny D: (outgroup, neognathus, tinamous, (rheas, (ostrich, (moas, CEK)))). Species considered are: *Little spotted (LS), Great spotted (GS), and Brown (B) kiwi; Cassowary; Emu; Eastern (E), Little Bush (LB), and Giant (G) moa; Ostrich, Lesser (L) and Greater (G) rhea; Talaupa (T), Giant (G),and Elegant Crested (EC) tinamou; Caiman; American Alligator; Brush (B) turkey; Chicken; Magpie (M) goose; Redhead duck; Little blue (L) penguin; Red-throated (RT) loon; Ruddy (R) Turnstone; Blackish (B) oystercatcher*

Then we study the MRCTs obtained for these real data with WO (trees displayed in Fig. 7). When we use ASAQ and we compute the MRCT with 2125, 5313, or all initial quartets (with both $m=1$ and $m=2$), we obtain the phylogeny

D: (outgroup, neognathus, tinamous, (rheas, (ostrich, (moas, CEK)))).

This phylogeny is also supported by a global NJ, although NJ splits tinamous and outgroup against the others. The same tree as NJ is obtained with SAQ (both for 2125 or 5313 initial quartets). Although the deepest interior branch lengths within ratites obtained by NJ are very small: (rheas, (ostrich, (moas, CEK):0.0025):0.003), SAQ and ASAQ give full support to these splits. Indeed, even when we use SAQ or ASAQ (with both $m=1$ or 2) with only 100 initial quartets, *all* 50 MRCTs share these splits. Unexpected trees are obtained by Erik+2, PL or 4P (either not solving correctly the neognathus clade, or not giving a CEK clade nor putting all tinamous in a clade).

### Execution Time and Implementation

An implementation of ASAQ in C++ can be found in

https://github.com/marinagarrote/ASAQ-method

The Q-methods WO, WIL and QP applied along the paper are implemented in

https://github.com/msabvid/weights_quartet_methods/

The computations on this paper have been performed on a computer with 6 Dual Core Intel(R) Xeon(R) E5-2430 Processor (2.20 GHz) equipped with 25 GB RAM running Debian GNU/Linux 8. We have used the g++ (Debian 4.9.2-10+deb8u2) 4.9.2 compiler and the C++ library for linear algebra & scientific computing *Armadillo* version 3.2.3 (Creamfields).

The average time required to compute ASAQ and PL weights for 100 alignments of 4 taxa and length 10 000 bp is 8.7 and 7.8 seconds, respectively. For each alignment, ML was stopped if it did not converge for a 4-tuple after 10 s; the frequency of convergence can be seen on Tables 9 and 11, 12, 13 and 14. The average time to reconstruct a CD tree given the weights for its 495 quartet subtrees is 4 seconds for WO, 89.5 seconds for WIL and 1.8 seconds for QP.

## Discussion

Via experiments on the simulation framework proposed by Ranwez and Gascuel (2001) but considering more general models of nucleotide substitution (including the GM model and mixtures of distributions), we observe a huge improvement on the performance of Q-based methods when weights from ASAQ, SAQ, Erik+2 and PL methods are considered. In general, the highest success is obtained by WO with the weighting system of ASAQ or PL, or QFM with ASAQ input. The accuracy of these methods is compatible with a global NJ or ML and improves upon them in the presence of mixtures or long branches when using ASAQ as weighting system. Moreover, these weights also outperform weights obtained by ML, even when data are generated under a GTR model and ML is estimating the same model.

It is worth noting that in the previous studies of Q-methods by Ranwez and Gascuel (2001); John et al. (2003), only weights from ML and NJ were considered. The results in this paper validate QFM, wQFM, WO and WIL as successful phylogenetic reconstruction methods if their input is a system of reliable weights. Moreover, as ASAQ assumes the most general Markov model and can deal with mixtures, this opens the door to use Q-methods to data generated by complex models.

**Fig. 17:**
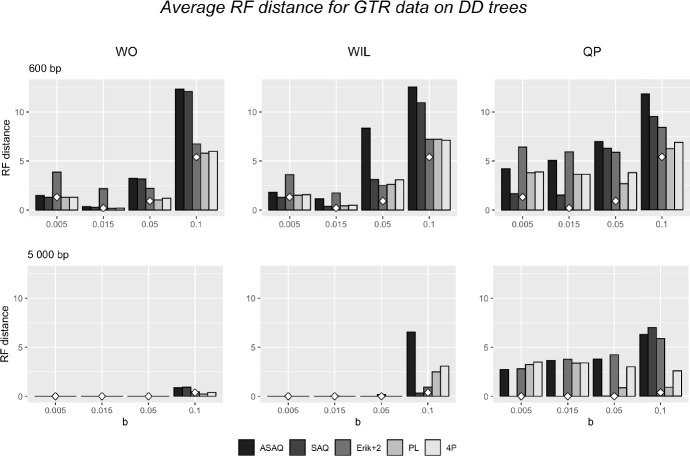
Average Robinson–Foulds distance for GTR data simulated on the tree *DD* with alignment length 600 bp (above) and 5 000 bp (below). The Q-methods WO (left), WIL (center) and QP (right) are applied with different systems of weights, namely ASAQ, SAQ, Erik+2, PL and 4P. The white diamonds represent the average RF distance of the tree reconstructed using a global NJ with paralinear distance. Specific values of these results are detailed in Table 11

We need to mention that the comparison performed against the ML weights (ML as input of Q-methods) or a global ML may not be totally fair because the model used in parameter estimation (continuous-time unrestricted model) does not fit the GM model used to simulate the data. It would certainly be interesting to develop maximum likelihood estimation based on the GM model and perform new tests. In any case, the results of ML weights are much worse than those of ASAQ, Erik+2 or PL also for GTR data (see Fig. 17 and Table 8 in the Appendix) and the results of a global ML are similar to a global NJ.

We have observed a good performance of input weights from ASAQ, Erik+2 and PL on simulated mixture data on 12-taxon trees, especially for 5 000 bp or more. ASAQ and PL are not likely to be statistically consistent for general mixtures on the same tree. Nevertheless, as Erik+2 is consistent on mixture data and PL is known to be consistent on some type of mixtures (see Allman et al. 2019), this suggests that ASAQ (being based on the accordance of Erik+2 and SAQ) might also be consistent on some types of mixture data, which would explain the good results obtained. We would like to point out that the mixture model allowed in Erik+2 (and hence in ASAQ) is actually more flexible than we mentioned. Indeed, the rank conditions considered in Erik+2 for quartets still hold if we let one cherry or one leaf evolve under a mixture with any number of categories (while the other cherry evolves under a single system of parameters), see Casanellas and Fernandez-Sanchez (2020). This makes the mixture model underlying Erik+2 and ASAQ more general, with implications in the mixture model that can be considered for Q-methods with input weights from ASAQ.

Our results on real data validate WO + ASAQ as a reliable method (both for yeast or ratites/tinamous data). Moreover, it is relevant to note that for the ratites/tinamou data, the topology obtained by WO + ASAQ or SAQ, or by NJ with paralinear distances does not agree with any of the topologies proposed in Phillips et al. (2009).

Note that the Q-methods considered here have higher order of computational complexity than NJ, but we have not yet explored the possibility of considering a subset of the possible 4-tuples as suggested in Snir and Rao (2010) or Davidson et al. (2018). It would be interesting to further explore these other versions of Q-methods with weights from ASAQ and PL, or even to restrict to quartets with highest weights as starting point for Q-methods.

On another direction, our results show that ASAQ is a powerful reconstruction method for quartet topology reconstruction. It assumes the most general model of nucleotide substitution (a general Markov model) of independently and identically distributed sites but it can also account for mixtures of distributions (with up to three categories). As it is based on the algebraic and semi-algebraic description of the model, it does not need to estimate the substitution parameters. In this sense, ASAQ could be easily adapted as a suitable method for dealing with amino acid substitutions as well. The incorporation of invariable sites seems also plausible via the results in Jayaswal et al. (2007); Steel et al. (2000). We plan to incorporate these features in a forthcoming version of the software.

As mentioned in the introduction, ASAQ is part of the set of phylogenetic reconstruction tools that are based on algebra. Most of these methods only reconstruct quartets because no statistically consistent (and computationally affordable) algebraic method for larger trees has been designed yet.

A study of ASAQ from a statistical point of view would certainly be relevant, as in this study the efficiency of the method has been solely based on the results obtained on large sets of simulated data.

## Figures and Tables

**Fig. 8:**
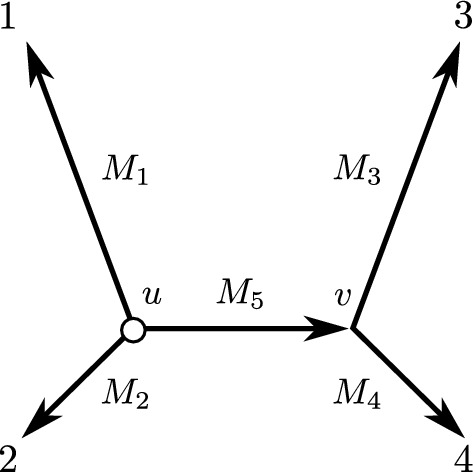
Markov process on the 12–34 quartet as in Theorem A.3**a**. The root is located at the interior node *u* close to the cherry (1, 2)

**Fig. 9:**
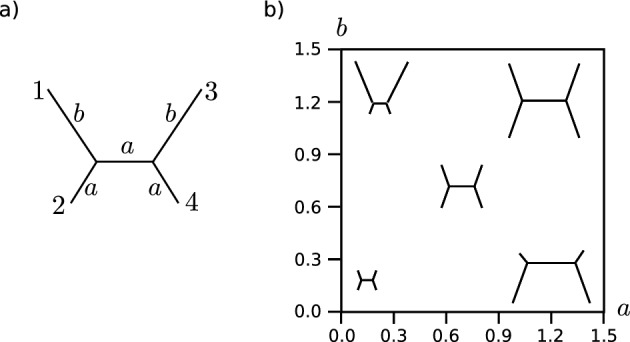
**a** 4-leaf tree where the length of two opposite branches and the interior branch are represented by *a*; the other two peripheral branches have length *b*. Branch lengths are measured as the expected number of substitutions per site. **b** Tree space considered for the tree in **a** where the branch lengths *a* and *b* are varied from 0.01 to 1.5 in steps of 0.02

**Fig. 10:**
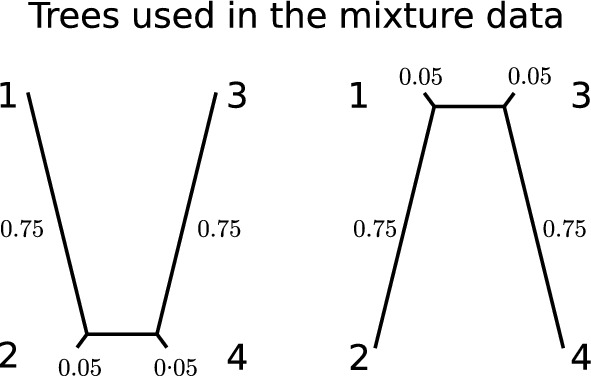
Trees considered in the simulations of mixture data: the alignments have two categories of the same size, each evolving under the GM model on one of the trees depicted above with the systems of branch lengths indicated. The internal branch length takes the same value in both categories and is varied from 0.01 to 0.4 in steps of 0.05

**Fig. 11:**
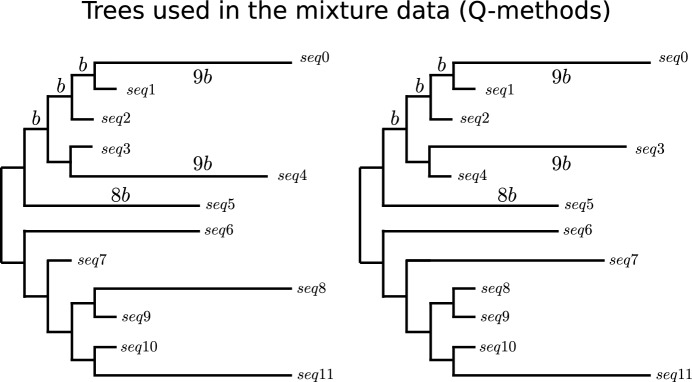
Trees considered in the simulations of mixture data for Q-methods: the alignment have two categories and the parameter $p\in \{0.25,0.50,0.75\}$ represents the proportion of the first category relative to the second. Data are generated under the GM model on one of the trees depicted above with the systems of branch lengths indicated for different values of *b*

**Fig. 12:**
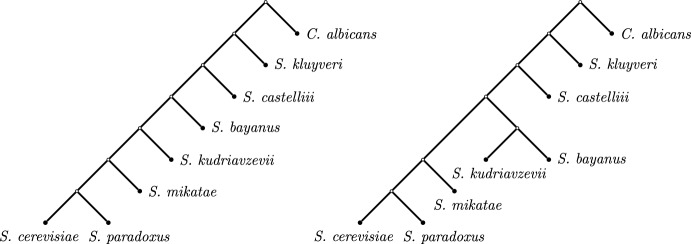
The tree *T* of Rokas et al. (2003) (left) and the alternative tree $T'$ of Phillips et al. (2004) (right) are constructed using the data provided by Jayaswal et al. (2014) with 42 337 s codon positions of 106 orthologous genes of *Saccharomyces cerevisiae*, *S. paradoxus*, *S. mikatae*, *S. kudriavzevii*, *S. castellii*, *S. kluyveri*, *S. bayanus* and *Candida albicans*

**Fig. 13:**
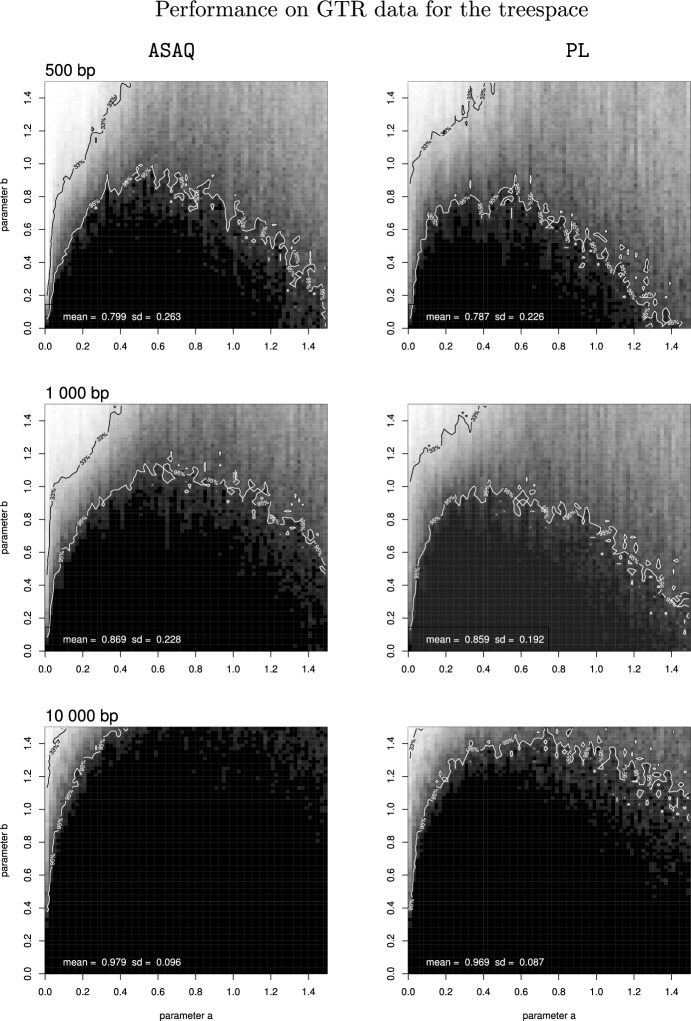
Performance of ASAQ (left) and PL (right) in the tree space of Fig. 9b on alignments of length 500 bp (top), 1 000 bp (middle) and 10 000 bp (bottom) generated under the GTR model. Black is used to represent 100% of successful quartet reconstruction, white to represent 0%, and different tones of gray the intermediate frequencies. The 95% contour line is drawn in white, whereas the 33% contour line is drawn in black

**Fig. 14:**
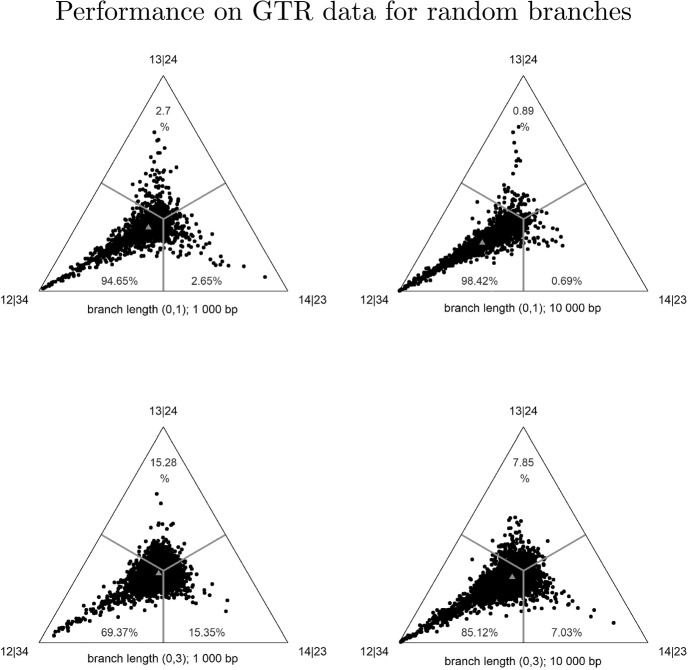
Ternary plots corresponding to the weights of ASAQ applied to 10000 alignments generated under the GTR model on the 12|34 tree. On each triangle the bottom-left vertex represents the underlying tree 12|34, the bottom-right vertex is the tree 13|24 and the top vertex is 14|23. The small gray triangle point depicted represents the average point of all the dots in the figure. Top: correspond to trees with random branch lengths uniformly distributed between 0 and 1; bottom: random branch lengths uniformly distributed between 0 and 3. Left: 1 000 bp; Right: 10 000 bp

**Fig. 15:**
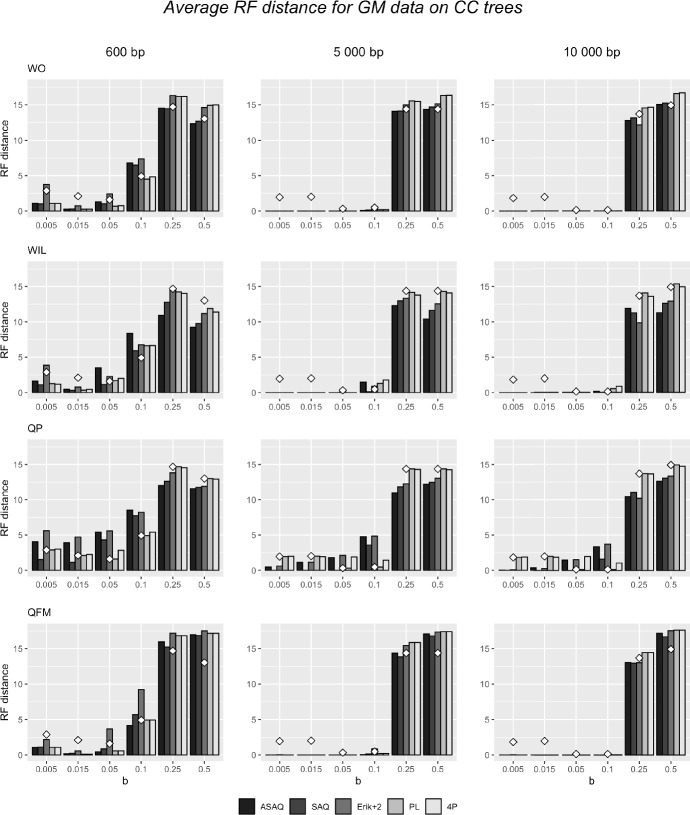
Average Robinson–Foulds distance for GM data simulated on the tree *CC* with alignment length 600 bp (left), 5 000 bp (center) and 10 000 bp (right). The Q-methods WO (first row), WIL (second row), QP (third row) and QFM (last row) are applied with different systems of weights, namely ASAQ, Erik+2, PL and 4P. The white diamond represent the average RF distance of the tree reconstructed using a global NJ with paralinear distance. Concrete values of these results are detailed in Table 6

**Fig. 16:**
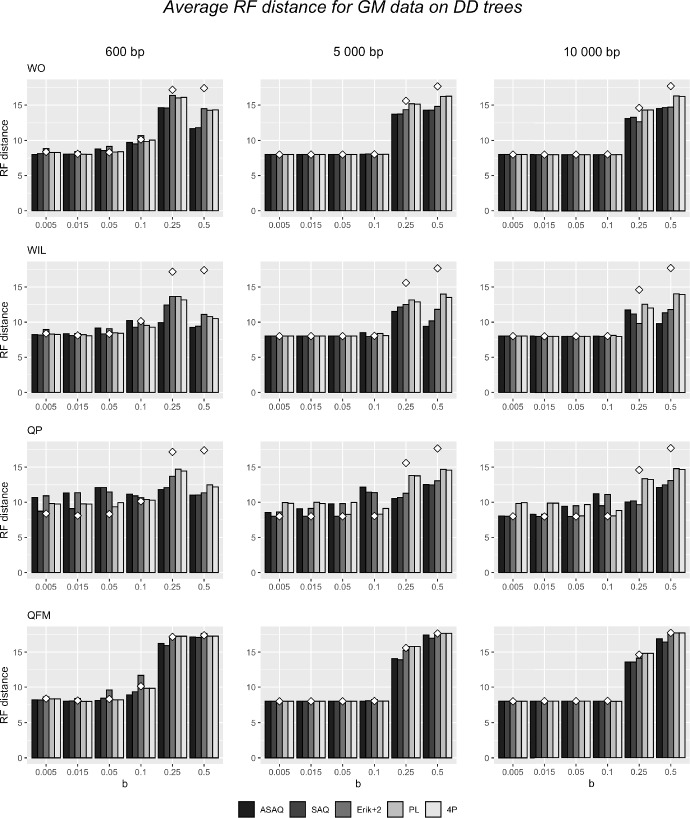
Average Robinson–Foulds distance for GM data simulated on the tree *DD* with alignment length 600 bp (left), 5 000 bp (center) and 10 000 bp (right). The Q-methods WO (first row), WIL (second row), QP (third row) and QFM (last row) are applied with different systems of weights, namely ASAQ, SAQ, Erik+2, PL and 4P. The white diamonds represent the average RF distance of the tree reconstructed using a global NJ with paralinear distance. Specific values of these results are detailed in Table 8

## A Weighting Systems and Technical Results About ASAQ

The underlying nucleotide substitution model we consider is a general Markov (GM) model (without treatment of indels or ambiguous sites). That is, we assume that substitution of nucleotides along a phylogenetic tree *T* follows a Markov process on *T* specified by a distribution $\pi$ of nucleotides at an interior node (which pays the role of the root) and $4\times 4$ Markov matrices $M^e$ for each edge *e* of *T*. We do not assume any extra condition on the transition matrices (neither stationarity nor time-reversibility).

As Markov matrices $M^e$ are not assumed of type $exp(t_eQ_e)$ (which would imply assuming a time-homogeneous continuous-time Markov process), the Markov process along each edge is not necessarily homogeneous through time (that is, the patterns of instantaneous mutation rates are allowed to change through time). Models that assume $M^e=exp(t_eQ_e)$ are *locally time-homogeneous*; this condition is very restrictive: for example less than 4% of diagonal largest-in-column Markov matrices are of this type (Casanellas et al. 2023). Moreover, if a Markov process on *T* assumes $M^e=exp(t_eQ)$ with the same instantaneous rate matrix *Q* for the whole tree (as is often assumed in phylogenetic reconstruction), the process is *globally time-homogeneous* in addition (i.e. instantaneous mutation rate patterns are constant throughout the tree). In this sense, the GM model underlying the methods Erik+2, SAQ, ASAQ, PL and 4P is *heterogeneous across lineages*.

### A.1 Weighting Systems

The input weights we consider in this paper are obtained from different quartet reconstruction methods: from SAQ, Erik+2 and ASAQ, from methods that use the paralinear distance (PL and 4P) and from maximum likelihood (ML). For any of these methods we describe here the weighting score used. To this end, let *f* be the vector of relative frequencies obtained from a DNA alignment of four taxa.

**Weights for methods based on the paralinear distance,** PL ** and** 4P **.** Consider two (ordered) nucleotide sequences $S_x$ and $S_y$ of the same length corresponding to two taxa *x* and *y*, respectively. Let *J* be the underlying joint probability matrix of $S_x$ and $S_y$, this is, the entry (*i*, *j*) of *J* is the probability (either theoretical or estimated by relative frequencies) of observing nucleotides *i* and *j* at the same position of $S_x$ and $S_y$ (so that the sum of entries in *J* is one). If $\det J\ne 0$ and all nucleotides are observed in $S_x$ and $S_y$, then the *paralinear distance* (Lake 1994) between *x* and *y* is

2 $$\begin{aligned} d_{x,y}=-\log \frac{|\det J|}{\sqrt{\det D_x} \sqrt{\det D_y}}, \end{aligned}$$

where $D_x$ and $D_y$ are the diagonal matrices whose diagonal entries are given by $J {\textbf{1}}$ and ${\textbf{1}}^t J$, respectively; if $\det J=0$, we take $d_{x,y}$ as infinity. Based on the work by Lake (1994) and Ranwez and Gascuel (2001), given the vector *f* of relative frequencies from a quartet alignment, in Section *Materials and Methods* of the main document, we defined

$$\begin{aligned} {p\ell }_{12|34}(f)=\min \{d_{1,3}+d_{2,4},d_{1,4}+d_{2,3}\}-d_{1,2}-d_{3,4} \end{aligned}$$

which represents twice the length of the interior edge of the quartet (see Theorem A.3(b) below). We define ${p\ell }_{13|24}(f)$ and ${p\ell }_{14|23}(f)$ similarly.

In Mihaescu et al. (2009), the authors propose a slightly different way of weighting the quartets using the paralinear distance:

$$\begin{aligned} \ell _{12|34}(f)=\frac{1}{4}(d_{1,3}+d_{1,4}+d_{2,3}+d_{2,4})-\frac{1}{2}(d_{1,2}+d_{3,4}), \end{aligned}$$

($\ell _{13|24}(f)$ and $\ell _{14|23}(f)$ are defined analogously). These are the measures that we use for what we call the 4P method (standing for “four-point condition”). In the same paper quoted above, the authors establish the (theoretical) equivalence between NJ and a consistent quartet-based method that uses these weights. Observe also that these weights are equivalent to ${p\ell }_{12|34}(f)$ for theoretical data.

In order to assign a non-negative score to each quartet, we define the normalized weights for PL and 4P as

$$\begin{aligned} w_{p\ell }(f)= & {} \frac{1}{\sum _i \exp ({p\ell }_{T_i}(f))} \left( \exp ({p\ell }_{T_1}(f)), \exp ({p\ell }_{T_2}(f)), \exp ({p\ell }_{T_3}(f)) \right) . \\ w_{4p}(f)= & {} \frac{1}{\sum _i \exp (\ell _{T_i}(f))} \left( \exp (\ell _{T_1}(f)), \exp (\ell _{T_2}(f)), \exp (\ell _{T_3}(f)) \right) . \end{aligned}$$

**Weights for maximum likelihood.**

We weighted quartet trees for the ML method using the likelihoods associated to the three trees $T_1, T_2, T_3$ as done by Ranwez and Gascuel (2001): if $l_{T_i}(f)$ is the likelihood for the tree $T_i$ (with the MLE parameters), then the normalized weights for the three quartets are defined as

$$\begin{aligned} w_{ml} (f)= \frac{1}{\sum _i l_{T_i}(p)} \left( l_{T_1}(f), l_{T_1}(f), l_{T_1}(f) \right) . \end{aligned}$$

**Weights for** Erik+2 **.**

Given a bipartition *ij*|*kl* and a distribution $f\in {\mathbb {R}}^{256}$, denote by $Flat_{ij|kl}(f)$ the flattening of *f* according to that bipartition, that is, $Flat_{ij|kl}(f)$ is the $16\times 16$ matrix whose $(x_ix_j,x_kx_l)$-entry is the coordinate of *f* that matches $x_ix_jx_kx_l$ in the convenient order. Then, $Flat_{ij|kl}(f)$ has rank $\le 4$, while the flattenings corresponding to the other two bipartitions have rank 16 if the parameters are generic (Allman and Rhodes 2007)

If $\delta _4(\cdot )$ stands for the distance to the space of matrices with rank $\le 4$, the method Erik+2 computes for each quartet tree *T* the value

3 $$\begin{aligned} e_T(f)=\frac{\delta _4(Flat_T^r(f))+\delta _4(Flat_T^c(f))}{2}, \end{aligned}$$

where $Flat_T^r$ and $Flat_T^c$ stand for the (normalized) matrices obtained by dividing the rows and columns of the flattening matrix $Flat_T$ by the sum of its entries, respectively (as a variation we can consider the distance $\delta _{4m}(\cdot )$ to rank 4*m* matrices when we deal with mixed data with $m\in \{1,2,3\}$ categories). The right quartet tree should be the one that gives the minimal value; this is the output of the method.

In order to have a normalized scoring system that allows us to compare and represent the output of $\texttt {Erik+2}$ for each quartet tree *T*, we consider the inverse of the value $e_T(f)$ and then divide but the sum of these scores for all three quartet trees:

$$\begin{aligned} {\texttt {E}rik+2} (f)= \frac{1}{e}\left( e_{T_1}(f)^{-1},e_{T_2}(f)^{-1},e_{T_3}(f)^{-1}\right) \end{aligned}$$

where $$e:= e_{T_1}(f)^{-1} + e_{T_2}(f)^{-1} + e_{T_3}(f)^{-1}$$.

**Weights for** SAQ **.**

The method SAQ suggested by Casanellas et al. (2021b) associates the following score to the tree $T=12|34$:

$$\begin{aligned} s_T(f):=\sum _i \frac{\min \left\{ \delta _4(psd(Flat_{13|24}({\tilde{f}}_i))), \delta _4(psd(Flat_{1{4}|23}({\tilde{f}}_i)))\right\} }{\delta _4(psd(Flat_{12|34}({\tilde{f}}_i)))} \end{aligned}$$

where the sum runs over the sixteen 12|34 leaf-transformed distributions ${\tilde{f}}_i$ and *psd*(*M*) is the closest symmetric and positive semi-definite matrix to the matrix *M*. If *T* is any of the other two possible quartet trees, $s_T(f)$ is computed analogously by permuting the roles of the leaves accordingly. SAQ outputs the normalized three scores, that is, if $s:= s_{T_1}(f) + s_{T_2}(f) + s_{T_3}(f)$, then

$$\begin{aligned} {\texttt {S}AQ} (f):=\frac{1}{s} \left( s_{T_1}(f),s_{T_2}(f),s_{T_3}(f)\right) . \end{aligned}$$

### A.2 The Paralinear Distance and Theoretical Foundations of ASAQ

The following lemma generalizes the nonnegativity and the additivity properties of this dissimilarity map to general Markov matrices, extending the results of Lake (1994) to general Markov matrices as far as they are non-singular.

#### Lemma A.1

Let $\pi$ be the nucleotide distribution at *x* and consider a substitution process leading from *x* to *y* and ruled by a Markov matrix *M*. Then, the paralinear distance $d_{x,y}$ between *x* and *y* defined in (2) coincides with

4 $$\begin{aligned} d_{x,y}=-\log \frac{|\det M| \, \sqrt{\det D(\pi )}}{\sqrt{\det D(M^t \pi )}}, \end{aligned}$$

(where *D*(*u*) refers to the diagonal matrix whose diagonal entries are the coordinates of the vector *u*). Moreover, we have that

- $d_{x,y}\ge 0$, and the equality holds if and only if $M=Id$ or is a permutation matrix;
- the dissimilarity measure $d_{x,y}$ is additive.

#### Proof

We have that $J=D(\pi ) M$ is the underlying joint probability matrix between *x* and *y* so that the sum of its entries is one. Note that $\pi =J{\textbf{1}}$ and $\pi ^t M={\textbf{1}}^t J$, so $D_x=D(\pi )$ and $D_y=D(M^t \pi )$. Therefore,

$$\begin{aligned} d_{x,y}= & {} -\log \frac{|\det J|}{\sqrt{\det D_x}\sqrt{\det D_y}} = -\log \frac{|\det M| \, \det D_x}{\sqrt{\det D_x}\sqrt{\det D_y}}\\ {}= & {} -\log \frac{|\det M| \, \sqrt{\det D(\pi )}}{\sqrt{\det D(M^t \pi )}}. \end{aligned}$$

Now, we proceed to prove (a): $d_{x,y}\ge 0$ or equivalently, that

5 $$\begin{aligned} (\det M)^2 \; \frac{\det D(\pi )}{\det D(M^t\pi )} \le 1. \end{aligned}$$

First of all, since the matrix *M* is positive, we deduce that

$$\begin{aligned} \big |\det M \big |=\left| \sum _{\{i_1,i_2,i_3,i_4\}=[4]}\prod _j \; sgn(i_1,i_2,i_3,i_4) \, m_{i_j,j} \right| \le \sum _{\{i_1,i_2,i_3,i_4\}=[4]}\prod _j \;m_{i_j,j}. \end{aligned}$$

We have that

$$\begin{aligned} |\det M |\det D(\pi )= & {} |\det M | \,\prod _k \pi _k \\\le & {} \prod _k \pi _k \; \left( \sum _{\{i_1,i_2,i_3,i_4\}=[4]}\prod _j \;m_{i_j,j}\right) \\= & {} \sum _{\{i_1,i_2,i_3,i_4\} = [4]}\prod _j \; m_{i_j,j}\; \pi _j \\\le & {} \sum _{i_1,i_2,i_3,i_4 \in [4]}\prod _j \; m_{i_j,j}\, \pi _{i_j} = \prod _{j} \sum _i m_{ji} \pi _j = \prod _i (M^t\, \pi )_i \\= & {} \det D(M^t\pi ). \end{aligned}$$

By multiplying this inequality with $|\det M|\le 1$ (*M* is a Markov matrix), we obtain (5). Note that a similar argument is used in the proof of the main theorem in Steel (1994).

Note that if $d_{x,y}=0$, then necessarily $\det M=1$. Hadamard’s inequality implies that a Markov matrix *M* with determinant 1 is necessarily the identity matrix or a permutation matrix (Casanellas et al., 2022). Finally, the statement (b) follows easily from the expression (4). $\square$

Write $\Delta$ for the probability simplex in the space ${\mathbb {R}}^{256}$, that is, $\Delta$ is the set of all possible distribution vectors of patterns of nucleotides $p=(p_{x_1 x_2 x_3 x_4})_{x_1,x_2,x_3,x_4\in \{A,C,G,T\}}$. By Lemma 8 of Buneman (1971) applied to the paralinear distance, we get the following result (which can be also deduced almost directly from the definition (1) of the paper):

#### Lemma A.2

Assume that the determinant of every double marginalization of $p\in \Delta$ is non-zero (so that all values $d_{i,j}$, $i,j\in [4]$ can be computed). If $d_{i,j}\ge 0$ for all $i,j\in [4]$, then ${p\ell }_{A|B}(p)$ is strictly positive for at most one bipartition *A*|*B*.

Before proving the main theorem, we recall some definitions that will be needed. Following Sumner et al. (2008), given a vector $f=(f_{x_1,x_2,x_3,x_4})\in \mathbb {R}^{256}$ and $4\times 4$ Markov matrices $X_i, i=1,\ldots ,4$, the Markov action $(X_1,\ldots ,X_4)*f$ is the new vector $g=(g_{y_1,\ldots ,y_4})$ defined by

$$\begin{aligned} g_{y_1,\ldots ,y_4} = \sum _{x_1,\ldots ,x_4} f_{x_1,\ldots ,x_4} X_1(y_1,x_1) X_2(y_2,x_2)X_3(y_3,x_3)X_4(y_4,x_4). \end{aligned}$$

In case $f=p$ is the distribution vector arising from some tree *T* with certain transition matrices $\{M_i\}_i$, the new vector *g* corresponds to the distribution obtained by multiplying the original matrices at the pendant edges with the new matrices: $M_i \rightarrow M_i X_i$, $i=1,\ldots ,4$.

According to Sumner et al. (2017), a quartet inference method that assigns a triplet of weights *w*(*F*) to each array $F\in \mathbb {R}^{256}$ satisfies *the strong property II* if $w(X*F)=w(F)+(\lambda (X),\lambda (X),\lambda (X))$ for some additive function $\lambda$ on Markov matrices $X=(X_1,\ldots ,X_4)$. Essentially, the strong property II guarantees that if the probability parameters of the pattern distribution are affected by a linear operator, the effect on the output is that of adding the same quantity to each topology weight. Moreover, this quantity is consistent with the composition of operators.

Given an array *F*, for each pair $a,b\in [4], a< b$, we define matrices $N_{ab}$ by taking the (*i*, *j*)-entry as the double marginalization of *F* on the components different from *a* and *b*, where rows (respectively columns) are labeled by the states at *a* (resp. *b*). If $a>b$, then define $N_{ab} = N_{ba}^t$. For example, $(N_{13})_{i,j} = F_{i+j+}$ and $(N_{43})_{i,j} = F_{++ji}$.

#### Theorem A.3

Let *f* be the vector of relative frequencies of site patterns observed in an alignment of length *N* which has been generated according to a multinomial distribution with measure *p* (that is, *f* is the vector of relative frequencies that arises as *N* independent samples from *p*). Then we have

- $$\begin{aligned} \texttt{PL} (f)=({p\ell }_{12|34}(f),{p\ell }_{13|24}(f),{p\ell }_{14|23}(f)) \end{aligned}$$
  is a quartet inference method that satisfies the strong property II with $\lambda =0$. Moreover, if *p* arises from a Markov process on the tree $T=12|34$ of Fig. 8 with positive distribution $\pi$ at the root and invertible transition matrices, then the limit of the expectation of $\texttt{PL} (f)$ as *N* goes to infinity is
  $$\begin{aligned} \lim _{N\rightarrow \infty } {\mathbb {E}}(\texttt{PL} (f))= \left( {p\ell }_{12|34}(p),{p\ell }_{13|24}(p),{p\ell }_{14|23}(p) \right) ; \end{aligned}$$
- if *p* arises from a Markov process as in a), then ${p\ell }_{12|34}(p)=2 d_{u,v}\ge 0$ and ${p\ell }_{C|D}(p)=-{p\ell }_{12|34}(p)$ for any other bipartition $C|D \ne 12|34$;
- the paralinear method (which associates to a frequency vector *f* the tree $T_{A|B}$ with largest ${p\ell }_{A|B}(f)$) is statistically consistent for the general Markov model;
- ASAQ is statistically consistent for the general Markov model.

#### Proof

- The same proof of (Sumner et al. 2017, Theorem 3.1) applied to the paralinear distance gives that $\texttt {PL} (f)$ satisfies the strong property II with $\lambda =0$ in our case. Indeed, it is straightforward to see that ${p\ell }_{A|B}(f)$ is invariant by the Markov action: ${p\ell }_{A|B}((X_1,\dots ,X_4)*f)={p\ell }_{A|B}(f)$ for any $4\times 4$ Markov matrices $X_i$ and any bipartition *A*|*B*. This can be immediately seen by observing that for any array *F*
  $$\begin{aligned} {p\ell }_{A|B}(F)=-\log \, \max \left\{ \left| \frac{\det (N_{ac}) \det (N_{bd})}{\det (N_{ab}) \det (N_{cd})} \right| , \left| \frac{\det (N_{ad}) \det (N_{bc})}{\det (N_{ab}) \det (N_{cd})} \right| \right\} . \end{aligned}$$
  The claim about expectation follows by Taylor expansion of ${p\ell }_{A|B}(f)$ around the expectation of *f* (as ${\mathbb {E}}(f)=p$ componentwise).
- The first claim is a consequence of the additivity of the paralinear distance, see Lemma A.1. Indeed, under the assumption that *p* arises from the tree $T=12|34$, every quantity $d_{i,j}$ ($i,j\in [4]$) can be written as the sum of the paralinear distances attached to the edges of *T* between *i* and *j*. For example, $d_{1,3}=d_{1,u}+d_{u,v}+d_{v,3}$. Then the value ${p\ell }_{A|B}(p)$ results in $2 d_{u,v}$, which is nonnegative in virtue of (a) of Lemma A.1. Now, if *C*|*D* is a bipartition other than 12|34, then from (1) of the main document we immediately get ${p\ell }_{C|D}(p)=-{p\ell }_{12|34}(p)$.
- Denote $\delta = 2 d_{u,v}$. Then, following (*a*) we have that
  $$\begin{aligned} \!\lim _{N \rightarrow \infty } \!\texttt {PL} (f)\!=\!\!\lim _{N \rightarrow \infty }\!\left( {p\ell }_{12|34}(f),{p\ell }_{13|24}(f),{p\ell }_{14|23}(f)\!\right) \!=\! \texttt {PL} (p)\!=\!(\delta , -\delta , -\delta ), \end{aligned}$$
  where the last equality follows from (*b*). Therefore, for any $\epsilon >0$ there exist $N_1$, $N_2$, $N_3$ such that for any $N>N_0=\max \left\{ N_1,N_2,N_3\right\}$ we have
  $$\begin{aligned} \left| {p\ell }_{12|34}(f)-\delta \right|<\epsilon , \quad \left| {p\ell }_{13|24}(f)+\delta \right|<\epsilon , \quad \left| {p\ell }_{14|23}(f)+\delta \right| <\epsilon . \end{aligned}$$
  Then, for any $\epsilon <\delta$ we have
  $$\begin{aligned} {p\ell }_{12|34}(f)> \delta -\epsilon> 0> -\delta +\epsilon >\max \left\{ {p\ell }_{13|24}(f), {p\ell }_{14|23}(f)\right\} . \end{aligned}$$
  Then we can conclude that the method that chooses the tree *T* with largest ${p\ell }_{T}(f)$ chooses $T_{12|34}$ with probability tending to 1 when *N* tends to infinite.
- The statistical consistency of ASAQ follows from the statistical consistency of Erik+2 (Fernández-Sánchez and Casanellas 2016), SAQ (Casanellas et al. 2021b) and the paralinear method obtained in c). Indeed, if *f* is obtained from *N* samples of a multinomial distribution with measure *p* that has arisen from $T=T_{12|34}$ with non-singular parameters (positive distribution at the root and invertible transition matrices), then the probability that both methods (when applied to *f*) select $T_{12|34}$ tends to 1 when *N* tends to infinite.

$$\square $$

Note that under the assumption that $M_5$ is *diagonally largest in column* (DLC for short, see Chang (1996)), we deduce that if ${p\ell }_{A|B}(p)=0$, then $M_5$ is necessarily the identity matrix.

#### Remark A.4

In order to avoid numerical problems, we only compute ${p\ell }_{A|B}(f)$ if the condition number of the double marginal matrices (joint distributions at two leaves) involved in its computation are less than a certain tolerance. This is implemented using a parameter “threshold” set to 5000, but it can be modified by the user and can be adapted to the alignment length. If ${p\ell }_{A|B}(f)$ cannot be computed, then this is a sign of short sample size and ASAQ outputs the topology and the weighting system given by SAQ.

## B Tree figures

See Figs. 9, 10, 11 and 12.

## C Performance of ASAQ, PL and Q-Methods

### C.1 Performance of ASAQ and PL on the Treespace Under the GTR Model

Figure 13 represents the results of ASAQ and PL in recovering the correct quartet on data generated under the GTR model on the tree space described in *Simulated data for quartet reconstruction* in the Section on *Materials and Methods* of the main document.

### C.2 Performance of ASAQ on Quartets with Random Branch Lengths Under the GTR Model

Figure 14 shows the ternary plots corresponding to the ASAQ method applied to GTR data on trees with branches of random length.

### C.3 Performance of Q-Methods with Different Systems of Weights

Figures 15 and 16 represent the average Robinson–Foulds distance for GM data on *CC* and *DD* trees of Q-methods with ASAQ, Erik+2, PL and 4P weights. Similarly, Fig. 17 shows the average RF distance of Q-methods applied to GTR data on *DD* trees.

The results obtained by quartet puzzling, weight optimization and Willson methods applied to the input weights of the different methods on GTR data from the DD trees are shown in Fig. 17 (lengths 600 bp and 5 000 bp) and summarized in Table 6 (also including the results for 10 000 bp).

It is remarkable that in these simulations, it is enough to consider alignments of length 5 000 bp to obtain an almost perfect reconstruction of the original tree using the WO or Willson methods. It is also remarkable that for this particular topology the results described in *Unmixed data* in the Section *Results*, where the general Markov model was assumed, were not this good, obtaining distance values around 8 (see Fig. 16 ). In this case, assuming the GTR model these reconstruction methods manage to correctly infer the splits of the tree topology. Compared with these results, the performance of QP is poor, although it also improves the results obtained when applied to general Markov data. For short alignments (600 bp) PL and 4P weights obtained slightly better results overall than the other weights.

Note also the bad performance of WO when combined with ASAQ weights when applied to alignments of 600 bp and parameter $b=0.1$. For short alignments, ASAQ tends to choose the weights of SAQ, so this poor performance is probably caused by the bad results of SAQ when applied to GTR data (see Casanellas et al. (2021b) ).

The performance of every Q-method applied to ML weights for these data was quite poor and the resulting RF distance was never smaller than 8.95 (see Table 11).

### C.4 Tables

Table 5 reports the average number of cases in which $\texttt {Erik+2}$ and $\texttt {PL}$ results differ, and of those the proportion of times that $\texttt {SAQ}$ estimates the right topology. The figures show that among the three methods tested here, SAQ is the best option in all cases, except for alignments of length 10 000 bp generated under the GTR model.

Tables 6, 7 and 8 show the figures of the performance of the Q-methods QP, WIL, WO, wQFM and Qfm when applied to different weighting systems, namely ASAQ, SAQ, Erik+2, PL and 4P. Data have been simulated according to a general Markov model on the trees *CC*, *CD* and *DD*. The values in each table show the average of the *Robinson Foulds distance* from the original tree to the MRCT of 100 replicates for each of the 100 generated alignments. Similarly, Table 9 shows the results when the weighting system comes from ML. In case the reconstruction method failed to provide a weight configuration for some of the $$\left( {\begin{array}{c}12\\ 4\end{array}}\right) $$ quartets, a resulting tree cannot be constructed and the corresponding alignment has been neglected. The number in parentheses represents the number of consensus trees that we have been able to reconstruct. Table 10 shows the RF distances from the original tree to the tree constructed by applying global NJ with paralinear distances and global ML consistent with the 12.12 model.

In a similar way, Table 11 shows the RF distances for data generated under the GTR model. Finally, Tables 12, 13 and 14 show the RF distances on mixture data for different proportions between the two categories $p=0.25$, $p=0.5$ and $p=0.75$, respectively (see Sect. 2.2.2).

### C.5 Average Discordance Between $\texttt {Erik+2}$ and $\texttt {PL}$

**Table 5:** Average discordance between PL and Erik+2 for data generated on the tree 12|34 under the GM and GTR models, for different length alignments: 500, 1 000 and 10 000 bp (3rd column). The next three columns show for the cases when PL and Erik+2 do not agree the average success of each method (PL, Erik+2 or SAQ) to obtain the right topology

Model	Base pairs	$$\begin{array}{c} \% \text{ of } \text{ cases } \text{ when } \\ \texttt {PL} \ne \texttt {Erik+2} \end{array}$$	PL	Erik+2	SAQ
GM	500	26,08	57,77	31,67	70,29
GM	1000	20,65	60,35	30,81	71,38
GM	10000	3,73	41,99	53,32	63,32
GTR	500	26,44	52,43	33,10	58,88
GTR	1000	18,85	49,85	36,87	57,80
GTR	10000	3,44	12,65	79,36	43,12

#### C.5.1 Q-Methods on GM Data

**Table 6:** Average Robinson–Foulds distances for tree *CC*. Different combinations of Q-methods and weights (specified in the first two columns) have been applied to alignments with length 600 bp, 5 000 bp and 10 0000 bp evolving with different branch lengths

			Length 600 bp							Length 5 000 bp							Length 10 000 bp					
			b=0.005	0.015	0.05	0.1	0.25	0.5		0.005	0.015	0.05	0.1	0.25	0.5		0.005	0.015	0.05	0.1	0.25	0.5
WO	ASAQ		1.1	0.26	1.29	6.79	14.52	12.36		0	0	0	0.08	14.08	14.33		0	0	0	0	12.79	15.01
	SAQ		1	0.28	1.02	6.5	14.44	12.67		0	0	0	0.14	14.11	14.68		0	0	0	0	13.14	15.23
	Erik+2		3.75	0.74	2.42	7.37	16.29	14.61		0	0	0.1	0.65	14.98	15.12		0	0	0.04	0.1	12.18	15.08
	PL		1.06	0.26	0.69	4.5	16.19	14.92		0	0	0	0.19	15.52	16.32		0	0	0	0	14.54	16.57
	4P		1.06	0.26	0.76	4.84	16.16	14.96		0	0	0	0.19	15.45	16.35		0	0	0	0.02	14.65	16.68
WIL	ASAQ		1.61	0.47	3.5	8.37	10.92	9.23		0	0	0	1.47	12.31	10.41		0	0	0	0.14	11.91	11.29
	SAQ		1.1	0.3	1.14	5.93	12.79	9.77		0	0	0	0.12	12.97	11.63		0	0	0	0	11.26	12.62
	Erik+2		3.87	0.78	2.28	6.76	14.45	11.18		0	0	0.15	0.89	13.35	12.54		0	0	0.06	0.21	9.86	12.92
	PL		1.27	0.34	1.7	6.63	14.23	11.89		0	0	0	1.3	14.18	14.31		0	0	0	0.54	14.06	15.36
	4P		1.19	0.43	2	6.65	14.05	11.38		0	0	0	1.78	13.78	14.09		0	0	0	0.88	13.59	14.96
QP	ASAQ		4.03	3.89	5.41	8.52	12.03	11.56		0.47	1.11	1.81	4.76	10.94	12.24		0.05	0.35	1.46	3.34	10.44	12.63
	SAQ		1.53	1.11	4.31	7.74	12.63	11.77		0	0	0	3.59	11.85	12.49		0	0	0	1.59	11.04	13.05
	Erik+2		5.6	4.72	5.58	8.2	13.82	11.91		0.62	1.13	2.12	4.86	12.27	13.07		0.08	0.24	1.51	3.72	10.19	13.36
	PL		2.88	2.1	1.6	4.92	14.69	13.02		1.95	2.01	0.32	0.47	14.38	14.38		1.83	1.98	0.13	0.13	13.69	14.92
	4P		3	2.24	2.82	5.4	14.55	12.95		1.99	1.94	1.9	1.44	14.31	14.26		1.89	1.87	1.96	1.05	13.65	14.73
wQFM	ASAQ		1.08	0.36	4.52	10.14	14.98	16.70		0.00	0.00	0.00	2.80	14.42	16.68		0.00	0.00	0.00	0.00	13.66	16.68
	SAQ		1.10	0.26	0.62	6.40	15.68	16.68		0.00	0.00	0.00	0.08	15.18	16.72		0.00	0.00	0.00	0.00	13.88	16.46
	Erik+2		1.82	0.70	6.10	11.06	17.30	17.40		0.02	0.00	0.00	2.90	16.74	17.46		0.02	0.00	0.00	0.02	16.08	17.42
	PL		1.10	0.14	0.62	4.66	16.69	17.32		0.00	0.00	0.00	0.14	15.86	17.56		0.00	0.00	0.00	0.00	14.48	17.64
	4P		1.04	0.24	0.64	4.76	16.67	17.31		0.00	0.00	0.00	0.18	16.04	17.54		0.00	0.00	0.00	0.00	14.48	17.68
QFM	ASAQ		1.08	0.16	0.44	4.16	15.96	16.96		0.00	0.00	0.00	0.04	14.36	17.08		0.00	0.00	0.00	0.00	13.04	17.16
	SAQ		1.10	0.22	0.86	5.68	15.24	16.84		0.00	0.00	0.00	0.14	13.82	16.80		0.00	0.00	0.00	0.00	12.94	16.68
	Erik+2		2.16	0.58	3.68	9.22	17.18	17.52		0.02	0.00	0.00	0.86	15.42	17.36		0.02	0.00	0.00	0.02	13.00	17.54
	PL.4P		1.08	0.10	0.58	4.94	16.82	17.16		0.00	0.00	0.00	0.20	15.88	17.40		0.00	0.00	0.00	0.02	14.44	17.62

**Table 7:** Average Robinson–Foulds distances for tree *CD*. Different combinations of Q-methods and weights (specified in the first two columns) have been applied to alignments with length 600 bp, 5 000 bp and 10 0000 bp evolving with different branch lengths

			Length 600 bp							Length 5 000 bp							Length 10 000 bp					
			b=0.005	0.015	0.05	0.1	0.25	0.5		0.005	0.015	0.05	0.1	0.25	0.5		0.005	0.015	0.05	0.1	0.25	0.5
WO	ASAQ		4.4	4.22	4.84	8.46	14.47	11.83		4	4	4	4.06	13.43	14.26		4	4	4	4	12.69	14.9
	SAQ		4.5	4.18	4.58	8.37	14.54	11.95		4	4	4	4.14	13.34	14.73		4	4	4	4	12.68	15.2
	Erik+2		6.06	4.58	5.58	9.73	16.29	14.62		4	4	4.06	4.41	14.25	14.83		4	4	4	4	12.45	15.49
	PL		4.44	4.22	4.3	7.46	16.06	13.98		4	4	4	4.09	15.07	16.32		4	4	4	4	14.72	16.51
	4P		4.4	4.21	4.33	7.71	16.21	14.16		4	4	4	4.1	15.06	16.27		4	4	4	4	14.71	16.4
WIL	ASAQ		4.73	4.34	6.12	9.59	10.2	9.2		4	4	4	5.11	12.1	10		4	4	4	4.09	11.65	10.77
	SAQ		4.46	4.13	4.39	7.99	12.74	9.54		4	4	4	3.98	12.21	11.22		4	4	4	4	11.05	12.15
	Erik+2		6.1	4.59	5.53	8.38	13.89	10.98		4	4	4.04	4.63	12.82	11.94		4	4	4.02	4.05	10.15	12.42
	PL		4.69	4.33	4.69	8.08	14.17	11.07		4	4	4	4.68	13.64	14.25		4	4	4	4.36	13.32	14.85
	4P		4.64	4.36	4.97	8.17	13.84	10.79		4	4	4	4.85	13.57	14.04		4	4	4	4.42	13.05	14.36
QP	ASAQ		7.02	7.83	8.57	10.3	11.9	11.23		4.51	5.12	5.83	8.67	10.67	12.22		4.05	4.36	5.5	7.31	9.96	12.81
	SAQ		4.9	5.12	8.11	9.66	12.3	11.45		4	4	4	7.61	11.34	12.44		4	4	4	5.49	10.52	12.9
	Erik+2		8.17	8.06	8.77	9.65	13.83	11.64		4.66	5.07	5.94	8.1	11.97	13.1		4.04	4.32	5.56	7.27	9.79	13.54
	PL		6.12	6.06	5.4	8.05	14.24	12.43		5.9	5.89	4.39	4.46	14.19	14.56		5.95	5.9	4.16	4.09	13.5	14.9
	4P		6.15	6.12	6.17	8.25	14.32	12.23		5.93	5.97	5.9	5.36	13.94	14.55		5.88	5.99	5.89	4.97	13.52	14.66
wQFM	ASAQ		4.38	4.36	7.36	11.16	14.88	16.88		4.00	4.00	4.00	6.20	14.56	16.42		4.00	4.00	4.00	4.00	13.68	16.76
	SAQ		4.46	4.16	4.30	8.86	15.76	16.76		4.00	4.00	4.00	3.96	14.92	16.44		4.00	4.00	4.00	4.00	13.90	16.86
	Erik+2		5.18	4.54	8.64	11.96	17.32	17.52		4.04	4.00	4.00	6.10	16.58	17.54		4.02	4.00	4.00	3.98	16.22	17.62
	PL		4.42	4.22	4.36	7.70	17.06	17.07		4.00	4.00	4.00	4.10	15.44	17.60		4.00	4.00	4.00	4.00	14.86	17.68
	4P		4.42	4.18	4.32	7.72	17.05	17.06		4.00	4.00	4.00	4.12	15.34	17.60		4.00	4.00	4.00	4.00	14.82	17.74
QFM	ASAQ		4.38	4.16	4.20	6.84	16.22	16.92		4.00	4.00	4.00	4.00	14.04	16.86		4.00	4.00	4.00	4.00	13.28	17.28
	SAQ		4.42	4.20	4.46	7.74	15.42	16.82		4.00	4.00	4.00	4.10	13.42	16.64		4.00	4.00	4.00	4.00	12.86	16.74
	Erik+2		5.20	4.58	6.72	11.02	17.34	17.52		4.04	4.00	4.00	4.60	15.08	17.68		4.02	4.00	4.00	3.98	13.54	17.62
	PL.4P		4.46	4.18	4.28	7.80	17.00	17.12		4.00	4.00	4.00	4.08	15.38	17.54		4.00	4.00	4.00	4.02	14.64	17.66

**Table 8:** Average Robinson–Foulds distances for tree *DD*. Different combinations of Q-methods and weights (specified in the first two columns) have been applied to alignments with length 600 bp, 5 000 bp and 10 0000 bp evolving with different branch lengths

			Length 600 bp							Length 5 000 bp							Length 10 000 bp					
			b=0.005	0.015	0.05	0.1	0.25	0.5		0.005	0.015	0.05	0.1	0.25	0.5		0.005	0.015	0.05	0.1	0.25	0.5
WO	ASAQ		7.99	8.06	8.81	9.72	14.62	11.67		8	8	8	8.04	13.69	14.27		8	8	8	8	13.08	14.51
	SAQ		8.13	8.07	8.56	9.52	14.57	11.81		8	8	8	8.1	13.72	14.28		8	8	8	8	13.28	14.65
	Erik+2		8.85	8.43	9.17	10.7	16.37	14.49		8	8	7.96	8.12	14.34	14.81		8	8	7.98	7.94	12.64	14.73
	PL		8.3	8.04	8.35	9.83	16.01	14.26		8	8	8	8.02	15.19	16.22		8	8	8	8	14.3	16.31
	4P		8.3	8.02	8.4	10.05	16.1	14.32		8	8	8	8.03	15.12	16.25		8	8	8	8.01	14.31	16.25
WIL	ASAQ		8.21	8.34	9.16	10.24	9.93	9.24		8	8	8	8.49	11.53	9.4		8	8	8	8.02	11.75	9.81
	SAQ		8.16	8.08	8.32	9.26	12.44	9.41		8	8	8	7.94	12.14	10.19		8	8	8	8	11.16	11.34
	Erik+2		8.93	8.36	9.05	9.92	13.65	11.1		8	8	7.98	8.13	12.51	11.82		8	8	7.98	7.95	9.83	11.81
	PL		8.29	8.21	8.48	9.54	13.65	10.79		8	8	8	8.37	13.16	13.99		8	8	8	8.14	12.56	14.04
	4P		8.24	8.05	8.4	9.26	13.17	10.49		8	8	8	8.08	12.88	13.52		8	8	8	7.98	12.02	13.97
QP	ASAQ		10.68	11.34	12.09	11.16	11.83	11.01		8.53	9.07	9.77	12.17	10.51	12.54		8.06	8.29	9.45	11.24	10.06	12.13
	SAQ		8.72	9.09	12.09	10.91	12.08	11.03		8	8	8	11.46	10.68	12.47		8	8	8	9.54	10.21	12.51
	Erik+2		10.92	11.36	11.48	10.65	13.7	11.35		8.63	9.13	9.81	11.37	11.29	13.07		8.01	8.33	9.54	11.12	9.67	13.1
	PL		9.82	9.77	9.36	10.39	14.72	12.49		9.95	9.99	8.28	8.3	13.83	14.69		9.83	9.91	8.1	8.1	13.38	14.81
	4P		9.75	9.74	9.94	10.29	14.44	12.2		9.85	9.82	9.97	9.12	13.77	14.58		9.94	9.9	9.69	8.86	13.26	14.69
wQFM	ASAQ		8.12	8.16	10.80	11.90	15.84	17.06		8.00	8.00	8.00	10.10	14.96	17.04		8.00	8.00	8.00	8.00	14.70	16.34
	SAQ		8.08	8.02	8.26	10.34	16.48	16.90		8.00	8.00	8.00	7.94	14.70	17.02		8.00	8.00	8.00	8.00	14.32	16.44
	Erik+2		8.64	8.54	10.78	12.26	17.36	17.62		8.04	8.00	8.00	9.40	16.58	17.60		8.02	8.00	8.00	7.98	16.34	17.68
	PL		8.44	8.04	8.30	9.92	17.18	17.32		8.00	8.00	8.00	8.02	15.80	17.64		8.00	8.00	8.00	8.00	14.94	17.72
	4P		8.40	8.12	8.32	10.16	17.17	17.39		8.00	8.00	8.00	8.04	16.06	17.62		8.00	8.00	8.00	8.00	14.96	17.70
QFM	ASAQ		8.20	8.00	8.12	8.90	16.24	17.16		8.00	8.00	8.00	8.00	14.06	17.42		8.00	8.00	8.00	8.00	13.56	16.88
	SAQ		8.18	8.04	8.44	9.36	15.92	17.10		8.00	8.00	8.00	8.08	13.90	16.98		8.00	8.00	8.00	8.00	13.56	16.40
	Erik+2		8.64	8.40	9.60	11.68	17.28	17.66		8.04	8.00	8.00	8.26	15.22	17.72		8.02	8.00	8.00	7.98	14.12	17.66
	PL.4P		8.34	7.98	8.20	9.84	17.26	17.28		8.00	8.00	8.00	8.02	15.78	17.68		8.00	8.00	8.00	7.98	14.80	17.70

**Table 9:** Average Robinson–Foulds distance for WO, QP and WIL on trees *CC,CD,DD* when ML weights are used. Different combinations of Q-methods and weights (specified in the first two columns) have been applied to alignments with length 600 bp, 5 000 bp and 10 0000 bp evolving with different branch lengths. The numbers in parentheses represent the number of consensus trees that we have been able to reconstruct (as ML may not converge for some quartets and in this case we cannot reconstruct the tree); if it is missing, then we have been able to reconstruct all the trees

			Length 600 bp							Length 5 000 bp							Length 10 000 bp					
			b=0.005	0.015	0.05	0.1	0.25	0.5		0.005	0.015	0.05	0.1	0.25	0.5		0.005	0.015	0.05	0.1	0.25	0.5
CC	WO		12.71	12.25	12.75	11 (3)	–	–		11.03	11.18 (93)	11.83 (93)	– (0)	–	–		10.97	11.01 (96)	11.69 (91)	13 (1)	–	–
	WIL		9.83	9.79	9.84	9 (3)	–	–		9.97	9.28 (93)	9.27 (93)	– (0)	–	–		9.33	9 (96)	8.98 (91)	9 (1)	–	–
	QP		9.55	9.58	9.67	10.67 (3)	–	–		9	9.08 (93)	9.65 (93)	– (0)	–	–		9	9.11 (96)	9.6 (91)	9 (1)	–	–
CD	WO		12.28 (78)	12.04 (98)	12.32 (95)	10 (1)	–	–		11.04	11.18 (96)	11.76 (96)	10 (1)	–	–		10.96	10.96 (96)	11.55 (98)	9 (1)	–	–
	WIL		9.87 (78)	9.9 (98)	9.61 (95)	9 (1)	–	–		10	9.34 (96)	9.34 (96)	9 (1)	–	–		9.33	8.99 (96)	8.99 (98)	9 (1)	–	–
	QP		9.53 (78)	9.54 (98)	9.67 (95)	10 (1)	–	–		9	9.08 (96)	9.58 (96)	10 (1)	–	–		9	9.09 (96)	9.6 (98)	10 (1)	–	–
DD	WO		12.26 (74)	11.92 (93)	11.89 (89)	11 (8)	–	–		11.04	11.18 (85)	11.79 (87)	- (0)	–	–		10.98	10.91 (90)	11.52 (90)	- (0)	–	–
	WIL		9.88 (74)	9.72 (93)	9.6 (89)	9.38 (8)	–	–		10.04	9.31 (85)	9.36 (87)	- (0)	–	–		9.34	8.97 (90)	8.99 (90)	- (0)	–	–
	QP		9.47 (74)	9.6 (93)	9.73 (89)	9.62 (8)	–	–		9	9.11 (85)	9.59 (87)	- (0)	–	–		9	9.08 (90)	9.64 (90)	- (0)	–	–

**Table 10:** Average Robinson–Foulds distances for trees *CC,CD,DD* when reconstructed by a global NJ with paralinear distance and a global ML. Different combinations of Q-methods and weights (specified in the first two columns) have been applied to alignments with length 600 bp, 5 000 bp and 10 0000 bp evolving with different branch lengths

	Length	600 bp						5 000 bp						10 000 bp					
	b	0.005	0.015	0.05	0.1	0.25	0.5	0.005	0.015	0.05	0.1	0.25	0.5	0.005	0.015	0.05	0.1	0.25	0.5
CC	NJ	2.88	2.1	1.6	4.92	14.69	13.02	1.95	2.01	0.32	0.47	14.38	14.38	1.83	1.98	0.13	0.13	13.69	14.92
	ML	3	2.24	2.82	5.4	14.55	12.95	1.99	1.94	1.9	1.44	14.31	14.26	1.89	1.87	1.96	1.05	13.65	14.73
CD	NJ	4.42	4.2	4.34	7.7	17.28	17.41	4	4	4	4.12	15.84	17.5	4	4	4	4.04	14.8	17.38
	ML	4.36	4.04	4.10	5.06	5.72	17.82	4	4	4	4.68	14.48	17.78	4	4	4	4.8	14.8	17.78
DD	NJ	8.38	8.1	8.32	10.14	17.17	17.4	8	8	8	8.04	15.6	17.66	8	8	8	8.04	14.62	17.72
	ML	8.14	8	8.06	8.74	15.32	17.72	8	8	8	8.64	15	17.78	8	8	8	8.56	14.32	17.70

#### C.5.2 Q-Methods on GTR Data

**Table 11:** Average Robinson–Foulds distance for tree *DD* on GTR data. Different combinations of Q-methods and weights (specified in the first two columns) have been applied to alignments with length 600 bp, 5 000 bp and 10 0000 bp evolving with different branch lengths. The numbers in parentheses represent the number of consensus trees that we have been able to reconstruct (as ML may not converge for some quartets and in this case we cannot reconstruct the tree); if it is missing, then we have been able to reconstruct the 100 simulated trees

			Length 600 bp					Length 5 000 bp					Length 10 000 bp			
			b=0.005	0.015	0.05	0.1		0.005	0.015	0.05	0.1		0.005	0.015	0.05	0.1
WO	ASAQ		1.48	0.35	3.24	12.33		0	0	0	0.87		0	0	0	0
	SAQ		1.28	0.28	3.16	12.09		0	0	0	0.92		0	0	0	0
	Erik+2		3.87	2.19	2.21	6.74		0	0	0	0.46		0	0	0	0.02
	PL		1.29	0.16	1.03	5.8		0	0	0	0.22		0	0	0	0.04
	4P		1.3	0.19	1.2	5.99		0	0	0	0.4		0	0	0	0.12
	ML		12.71 (77)	12.45 (99)	11.98 (98)	- (0)		11.88 (64)	11.81 (86)	12.04 (97)	11.88 (33)		11.08 (71)	11.51 (85)	11.69 (96)	11.96 (47)
WIL	ASAQ		1.8	1.15	8.36	12.55		0	0	0	6.56		0	0	0	1.94
	SAQ		1.31	0.37	3.11	10.96		0	0	0	0.33		0	0	0	0
	Erik+2		3.61	1.74	2.5	7.23		0	0.02	0.16	0.93		0	0	0	0.21
	PL		1.52	0.42	2.63	7.23		0	0	0	2.49		0	0	0	1.47
	4P		1.57	0.48	3.08	7.12		0	0	0	3.07		0	0	0	1.77
	ML		9.71 (77)	9.57 (99)	9.43 (98)	- (0)		9.6 (64)	9.29 (86)	9.38 (97)	9.3 (33)		9.11 (71)	8.95 (85)	9 (96)	9.15 (47)
QP	ASAQ		4.21	5.05	6.98	11.83		2.73	3.65	3.8	6.31		2.74	3.74	4.03	4.47
	SAQ		1.67	1.53	6.3	9.52		0	0	0.02	6.99		0	0	0	4.18
	Erik+2		6.43	5.93	5.89	8.42		2.81	3.77	4.23	5.86		2.73	3.63	4.15	4.96
	PL		3.8	3.64	2.69	6.24		3.24	3.37	0.87	0.91		3.34	3.37	0.46	0.34
	4P		3.88	3.64	3.82	6.88		3.49	3.41	3.01	2.59		3.38	3.44	3.14	1.79
	ML		10.1 (77)	9.88 (99)	9.65 (98)	- (0)		9.19 (64)	9.28 (86)	9.28 (97)	9.82 (33)		9.1 (71)	9.11 (85)	9.32 (96)	9.38 (47)
Global NJ			1.32	0.2	0.92	5.4		0	0	0	0.38		0	0	0	0

#### C.5.3 Q-Methods on Mixture Data

**Table 12:** Average Robinson–Foulds distance on mixture data for $p=0.25$. The numbers in parentheses represent the number of consensus trees that we have been able to reconstruct (as ML may not converge for some quartets and in this case we cannot reconstruct the tree); if it is missing, then we have been able to reconstruct the 100 simulated trees. Figure 6 of the main document represents the bar plots of these values

			Length 600 bp					Length 5 000 bp					Length 10 000 bp			
			b=0.005	015	0.05	0.1		0.005	0.015	0.05	0.1		0.005	0.015	0.05	0.1
WO	ASAQ		1.74	0.46	1.73	6.68		0	0	0	0.95		0	0	0.02	1.31
	ASAQ (m=2)		1.63	0.48	1.24	5.8		0	0	0	0.96		0	0	0.02	0.98
	Erik+2 (m=2)		3.49	1.36	2.1	6.94		0.06	0.02	0.04	0.73		0.02	0	0.04	0.58
	PL		1.72	0.16	1.22	5.13		0	0	0.04	1.64		0	0	0.06	1.65
	ML		12.84	12.1	11.84	12 (1)		11.32	11.21	11.29	10 (1)		11	11.07	11.27	10.75 (4)
WIL	ASAQ		1.96	0.96	3.14	7.46		0	0	0.3	2.3		0	0	0.16	1.92
	ASAQ (m=2)		3.04	0.88	2.26	6.88		0	0	0.35	2.3		0	0	0.19	2.49
	Erik+2 (m=2)		4	1.57	2.71	6.98		0.04	0.08	0.08	1.3		0.02	0	0.06	0.83
	PL		1.83	0.41	1.8	6.67		0.02	0	0.29	2.81		0	0	0.31	2.81
	ML		10.07	9.57	9.49	9 (1)		9.95	9.53	9.32	10 (1)		9.24	8.98	9.03	9 (4)
QP	ASAQ		3.91	4.01	5.03	7.95		1.55	2.08	2.93	5.11		0.88	1.43	2.54	4.47
	ASAQ (m=2)		1.93	3.7	4.59	7.48		0.88	2.24	2.59	5.02		0.96	1.76	2.47	4.7
	Erik+2 (m=2)		3.54	3.23	4.19	7.36		0.14	0.63	2.33	4.33		0.04	0.08	1.52	3.69
	PL		3.52	3.02	3.37	6.12		2.81	2.61	2.57	3.11		2.77	2.6	2.58	2.95
	ML		9.66	10.24	9.65	9 (1)		9.11	9.29	9.87	9 (1)		9.28	9.15	9.8	10.25 (4)
Global NJ			1.84	0.16	0.96	4.98		0	0	0.06	1.68		0	0	0.04	1.64

**Table 13:** Average Robinson–Foulds distance on mixture data for $p=0.50$. The numbers in parentheses represent the number of consensus trees that we have been able to reconstruct (as ML may not converge for some quartets and in this case we cannot reconstruct the tree); if it is missing, then we have been able to reconstruct the 100 simulated trees. Figure 6 of the main document represents the bar plots of these values

			Length 600 bp					Length 5 000 bp					Length 10 000 bp			
			b=0.005	0.015	0.05	0.1		0.005	0.015	0.05	0.1		0.005	0.015	0.05	0.1
WO	ASAQ		2.95	0.86	1.83	5.58		0	0	0.16	1.81		0	0	0.2	2.28
	ASAQ (m=2)		3	0.86	1.3	4.89		0	0	0.1	1.24		0	0	0.07	1.45
	Erik+2 (m=2)		5.08	2.56	2.57	6.29		0.1	0	0.1	1.08		0.02	0	0.02	0.42
	PL		2.96	0.82	1.5	6.15		0	0	0.21	1.88		0	0	0.16	2.01
	ML		12.18	12.13	12.57	11.67 (3)		11.18	11.28	11.64	11 (1)		11.1	11.15	11.37	11 (1)
WIL	ASAQ		3.29	1.43	3.46	6.27		0	0	0.8	3.27		0	0	0.7	2.97
	ASAQ (m=2)		3.99	1.05	2.92	6.3		0.04	0	0.67	3.5		0	0	0.57	3.16
	Erik+2 (m=2)		5.3	2.55	3.17	6.58		0.1	0.02	0.25	1.7		0	0	0.02	0.85
	PL		3.28	0.99	2.69	7.31		0	0	0.66	3.36		0	0	0.41	3.16
	ML		9.85	9.67	9.74	9 (3)		9.82	9.33	9.4	9 (1)		9.19	9.11	9	9 (1)
QP	ASAQ		4.56	4.46	5.15	7.57		1.43	2.35	3.28	5.75		0.9	1.53	3.24	5.85
	ASAQ (m=2)		3	4.04	4.85	7.06		0.97	2.37	2.77	5.4		0.76	1.77	2.62	4.99
	Erik+2 (m=2)		4.89	3.88	4.54	7.21		0.13	0.92	2.52	4.76		0.02	0.16	1.6	4.04
	PL		4.29	3.36	3.52	6.44		2.75	2.77	2.56	3.57		2.83	2.71	2.72	3.37
	ML		9.67	9.89	9.7	10 (3)		9.25	9.38	10.07	10 (1)		9.13	9.17	9.63	9 (1)
Global NJ			2.98	0.82	1.18	6.18		0	0	0.18	1.84		0	0	0.14	2.14

**Table 14:** Average Robinson–Foulds distance on mixture data for $p=0.75$. The numbers in parentheses represent the number of consensus trees that we have been able to reconstruct (as ML may not converge for some quartets and in this case we cannot reconstruct the tree); if it is missing, then we have been able to reconstruct the 100 simulated trees. Figure 6 of the main document represents the bar plots of these values

			Length 600 bp					Length 5 000 bp					Length 10 000 bp			
			b=0.005	0.015	0.05	0.1		0.005	0.015	0.05	0.1		0.005	0.015	0.05	0.1
WO	ASAQ		1.82	0.87	1.57	6.27		0	0	0	1.44		0	0	0.12	1.54
	ASAQ (m=2)		1.76	0.44	0.95	5.55		0	0	0.04	1.01		0	0	0.08	0.81
	Erik+2 (m=2)		4.62	1.7	2.06	6.87		0.06	0	0.04	1.08		0	0.02	0	0.28
	PL		1.61	0.56	1.09	5.22		0	0	0.14	1.36		0	0	0.02	1.5
	ML		12.81	12.2	12.15	- (0)		12.11	11.43	11.68	- (0)		11.25	11.28	11.41	- (0)
WIL	ASAQ		2.07	0.99	3.63	7.9		0	0	0.16	3.12		0	0	0.32	2.87
	ASAQ (m=2)		2.87	0.77	2.56	7.15		0	0	0.3	3.21		0	0	0.22	2.93
	Erik+2 (m=2)		5.12	1.74	2.41	6.75		0.02	0.01	0.07	1.45		0	0.02	0.01	0.33
	PL		1.82	1	2.2	6.56		0	0	0.6	2.97		0	0	0.33	3.07
	ML		9.81	9.89	9.36	- (0)		9.94	9.53	9.46	- (0)		9.09	8.86	9.03	- (0)
QP	ASAQ		4.15	4.35	4.97	8.13		1.39	1.99	2.91	5.47		0.73	1.49	2.73	5.63
	ASAQ (m=2)		1.88	4.02	5.09	7.77		0.88	2.36	2.77	5.34		0.81	1.83	2.46	5
	Erik+2 (m=2)		4.35	3.08	4.24	7.36		0.1	0.58	2.52	4.81		0	0.04	1.98	4.05
	PL		3.63	3.42	3.55	5.77		2.52	2.72	2.75	2.95		2.6	2.65	2.63	3.12
	ML		9.88	9.67	9.64	- (0)		9.06	9.2	10.14	- (0)		9.06	9.19	9.73	- (0)
Global NJ			1.46	0.6	1.12	5.4		0	0	0.12	1.46		0	0	0.04	1.74

## Footnotes

1 under the name “SplitSup” in http://github.com/eallman/SplitSup/.

## References

[CR1] Abadi S, Azouri D, Pupko T, Mayrose I (2019) Model selection may not be a mandatory step for phylogeny reconstruction. Nat Commun 10:934 10.1038/s41467-019-08822-w PMC6389923 30804347

[CR2] Allman ES, Banos H, Rhodes JA (2022). Identifiability of species network topologies from genomic sequences using the logdet distance. J Math Bio.

[CR3] Allman ES, Rhodes JA, Gascuel O, Steel MA (2007). Phylogenetic invariants. Reconstructing evolution.

[CR4] Allman ES, Rhodes JA, Taylor A (2014). A semialgebraic description of the general Markov model on phylogenetic trees. SIAM J Discret Math.

[CR5] Allman ES, Kubatko LS, Rhodes JA (2016). Split scores: a tool to quantify phylogenetic signal in genome-scale data. Syst Biol.

[CR6] Allman ES, Baños H, Rhodes JA (2019). NANUQ: a method for inferring species networks from gene trees under the coalescent model. Algorithms Mol Biol.

[CR7] Allman ES, Long C, Rhodes JA (2019). Species tree inference from genomic sequences using the logdet distance. SIAM J Appl Algebr Geom.

[CR8] Benito J, Kuo P-C, Widrig KE, Jagt JWM, Field DJ (2022). Cretaceous ornithurine supports a neognathous crown bird ancestor. Nature.

[CR9] Buneman P (1971) The recovery of trees from measures of dissimilarity. In: Mathematics in the archaeological and historical sciences, pp 387–395

[CR10] Casanellas M, Fernández-Sánchez J, Garrote-López M (2021). Distance to the stochastic part of phylogenetic varieties. J Symb Comput.

[CR11] Casanellas M, Fernández-Sánchez J, Garrote-López M (2021). SAQ: semi-algebraic quartet reconstruction method. IEEE/ACM Trans Comput Biol Bioinf.

[CR12] Casanellas M, Fernández-Sánchez J, Roca-Lacostena J (2023). The embedding problem for Markov matrices. Publicacions Matemàtiques.

[CR13] Casanellas M, Fernández-Sánchez J (2021) Rank conditions on phylogenetic networks. In: Extended abstracts GEOMVAP 2019. Trends in mathematics, vol. 15. Springer-Birkhäuser, pp 65–69

[CR14] Chifman J, Kubatko LS (2014). Quartet inference from SNP data under the coalescent model. Bioinformatics.

[CR15] Davidson R, Lawhorn M, Rusinko J, Weber N (2018). Efficient quartet representations of trees and applications to supertree and summary methods. IEEE/ACM Trans Comput Biol Bioinf.

[CR16] Felsenstein J (2004). Inferring phylogenies.

[CR17] Fernández-Sánchez J, Casanellas M (2016). Invariant versus classical approach when evolution is heterogeneous across sites and lineages. Sys Bio.

[CR18] Fernández-Sánchez J, Sumner JG, Jarvis PD, Woodhams MD (2015). Lie Markov models with purine/pyrimidine symmetry. J Math Biol.

[CR19] Garrote-López M (2021) Algebraic and semi-algebraic phylogenetic reconstruction. Phd. thesis, Universitat Politècnica de Catalunya. https://upcommons.upc.edu/handle/2117/351096

[CR20] Gascuel O (1994). A note on Sattath and Tversky’s, Saitou and Nei’s, and Studier and Keppler’s algorithms for inferring phylogenies from evolutionary distances. Mol Biol Evolut.

[CR21] Holland BR, Huber KT, Moulton V, Lockhart PJ (2004). Using consensus networks to visualize contradictory evidence for species phylogeny. Mol Biol Evolut.

[CR22] Holland BR, Jarvis PD, Sumner JG (2012). Low-parameter phylogenetic inference under the general Markov model. Syst Biol.

[CR23] Huelsenbeck JP (1995). Performance of phylogenetic methods in simulation. Syst Biol.

[CR24] Jayaswal V, Robinson J, Jermiin LS (2007). Estimation of phylogeny and invariant sites under the general Markov model of nucleotide sequence evolution. Syst Biol.

[CR25] Jayaswal V, Wong TKF, Robinson J, Poladian L, Jermiin LS (2014). Mixture models of nucleotide sequence evolution that account for heterogeneity in the substitution process across sites and across lineages. Syst Biol.

[CR26] Jermiin LS, Catullo RA, Holland BR (2020). A new phylogenetic protocol: dealing with model misspecification and confirmation bias in molecular phylogenetics. NAR Genom Bioinform.

[CR27] John SK, Warnow T, Moret BM, Vawter L (2003). Performance study of phylogenetic methods: (unweighted) quartet methods and neighbor-joining. J Algorithms.

[CR28] Kaehler BD, Yap VB, Zhang R, Huttley GA (2015). Genetic distance for a general non-stationary Markov substitution process. Syst Biol.

[CR29] Kedzierska AM, Casanellas M (2012). GenNon-h: generating multiple sequence alignments on nonhomogeneous phylogenetic trees. BMC Bioinform.

[CR30] Kolaczkowski B, Thornton JW (2004). Performance of maximum parsimony and likelihood phylogenetics when evolution is heterogeneous. Nature.

[CR31] Lake JA (1994). Reconstructing evolutionary trees from DNA and protein sequences: paralinear distances. Proc Natl Acad Sci.

[CR32] Mahbub M, Wahab Z, Reaz R, Rahman MS, Bayzid MS (2021). wQFM: highly accurate genome-scale species tree estimation from weighted quartets. Bioinformatics.

[CR33] Mihaescu R, Levy D, Pachter L (2009). Why neighbor-joining works. Algorithmica.

[CR34] Minh BQ, Schmidt HA, Chernomor O, Schrempf D, Woodhams MD, von Haeseler A, Lanfear R (2020). IQ-TREE 2: new models and efficient methods for phylogenetic inference in the genomic era. Mol Biol Evolut.

[CR35] Paradis E, Claude J, Strimmer K (2004). APE: analyses of phylogenetics and evolution in R language. Bioinformatics.

[CR36] Paton T, Haddrath O, Baker AJ (2002). Complete mitochondrial DNA genome sequences show that modern birds are not descended from transitional shorebirds. Proc Biol Sci.

[CR37] Phillips MJ, Delsuc F, Penny D (2004). Genome-scale phylogeny and the detection of systematic biases. Mol Biol Evolut.

[CR38] Phillips MJ, Gibb GC, Crimp EA, Penny D (2009). Tinamous and Moa Flock together: mitochondrial genome sequence analysis reveals independent losses of flight among ratites. Syst Biol.

[CR39] Rambaut A, Grass NC (1997). Seq-Gen: an application for the Monte Carlo simulation of DNA sequence evolution along phylogenetic trees. Bioinformatics.

[CR40] Ranwez V, Gascuel O (2001). Quartet-based phylogenetic inference: improvements and limits. Mol Biol Evolut.

[CR41] Reaz R, Bayzid MS, Rahman MS (2014). Accurate phylogenetic tree reconstruction from quartets: a heuristic approach. PLoS ONE.

[CR42] Robinson D, Foulds L (1981). Comparison of phylogenetic trees. Math Biosci.

[CR43] Rokas A, Williams BL, King N, Carroll SB (2003). Genome-scale approaches to resolving incongruence in molecular phylogenies. Nature.

[CR44] Rusinko J, Hipp B (2012). Invariant based quartet puzzling. Algorithms Mol Biol.

[CR45] Schmidt HA, Strimmer K, Vingron M, von Haeseler A (2002). Tree-puzzle: maximum likelihood phylogenetic analysis using quartets and parallel computing. Bioinformatics.

[CR46] Snir S, Rao S (2010). Quartets MaxCut: a divide and conquer quartets algorithm. IEEE/ACM Trans Comput Biol Bioinf.

[CR47] Steel MA, Huson D, Lockhart PJ (2000). Invariable sites models and their use in phylogeny reconstruction. Syst Biol.

[CR48] Strimmer K, von Haeseler A (1996). Quartet puzzling: a quartet maximum-likelihood method for reconstructing tree topologies. Mol Biol Evolut.

[CR49] Strimmer K, von Haeseler A (1997). Likelihood-mapping: A simple method to visualize phylogenetic content of a sequence alignment. Proc Natl Acad Sci.

[CR50] Strimmer K, Goldman N, von Haeseler A (1997). Bayesian probabilities and quartet puzzling. Mol Biol Evolut.

[CR51] Sukumaran J, Holder MT (2010). DendroPy: a Python library for phylogenetic computing. Bioinformatics.

[CR52] Sumner JG, Charleston MA, Jermiin LS, Jarvis PD (2008). Markov invariants, plethysms, and phylogenetics. J Theor Biol.

[CR53] Sumner JG, Taylor A, Holland BR, Jarvis PD (2017). Developing a statistically powerful measure for quartet tree inference using phylogenetic identities and Markov invariants. J Math Biol.

[CR54] Swofford DL (2003) PAUP$${}^\ast $$: Phylogenetic analysis using parsimony ($${}^\ast $$and Other Methods), Version 4.0b10. Sinauer Associates, Sunderland, Massachusetts

[CR55] Vera-Ruiz VA, Robinson J, Jermiin LS (2021). A likelihood-ratio test for lumpability of phylogenetic data: is the Markovian property of an evolutionary process retained in recoded DNA?. Syst Biol.

[CR56] Willson SJ (1999). Building phylogenetic trees from quartets by using local inconsistency measures. Mol Biol Evol.

[CR57] Yang Z (1997). PAML: a program package for phylogenetic analysis by maximum likelihood. Bioinformatics.

[CR58] Yu Y, Than C, Degnan JH, Nakhleh L (2011). Coalescent histories on phylogenetic networks and detection of hybridization despite incomplete lineage sorting. Syst Biol.

[CR59] Zou L, Susko E, Field C, Roger AJ (2012). Fitting nonstationary general-time-reversible models to obtain edge-lengths and frequencies for the Barry-Hartigan model. Syst Biol.

[CR60] Zou Z, Zhang H, Guan Y, Zhang J (2019). Deep residual neural networks resolve quartet molecular phylogenies. Mol Biol Evol.

